# The First Step is the Hardest: A Mixed Methods Single-Case Experimental Design Study of a VR-Enhanced Training Program in a Forensic Youth Care Setting

**DOI:** 10.1007/s10802-025-01313-1

**Published:** 2025-04-14

**Authors:** Renée E. Klein Schaarsberg, Nicole Klinkhamer, Levi van Dam, Samantha Bouwmeester, Ramón J. L. Lindauer, Arne Popma

**Affiliations:** 1 Child and Adolescent Psychiatry & Psychosocial Care, Amsterdam UMC, Vrije Universiteit Amsterdam, Amsterdam, The Netherlands; 2 Department of Child Development and Education, University of Amsterdam, Amsterdam, The Netherlands; 3 Dutch Innovation Network for Societal Youth Challenges, Garage2020, Amsterdam, The Netherlands; 4 Child and Adolescent Psychiatry, Amsterdam UMC, University of Amsterdam, Amsterdam, The Netherlands; 5 Levvel, Academic Center for Child and Adolescent Psychiatry, Amsterdam, The Netherlands; 6 Amsterdam Public Health, Mental Health, Amsterdam, The Netherlands; 7 Out of the Boxplot, Rotterdam, The Netherlands; 8 Department of Developmental Psychology, Tilburg School of Behavioral Sciences, Tilburg University, Tilburg, The Netherlands

**Keywords:** Mentalization, Virtual reality, Single-case experimental design, Adolescence, Forensic youth care

## Abstract

Street Temptations is a virtual reality-enhanced training program developed as an add-on intervention for adolescents with disruptive behavior problems. Using mentalization as a primary treatment mechanism, the program aims to address cognitive distortions and increase motivation for behavior change. This study provides an initial evaluation of Street Temptations in a forensic youth care setting, utilizing both quantitative and qualitative data within an A-B-A’ single-case experimental design. Throughout a baseline, intervention, and follow-up phase, adolescents completed daily measurements on cognitive distortions and motivation for behavior change. Secondary outcomes were assessed before baseline, after intervention, and after follow-up, which included mentalization and perspective-taking. Qualitative data were collected after the intervention through separate interviews with adolescents and Street Temptations therapists. A total of 8 adolescents were included in the study, 5 of whom dropped out, leaving 3 who provided sufficient data for visual inspection of the outcomes. The observed patterns suggested an increase in adolescents’ awareness of others’ perspectives during participation in Street Temptations, potentially indicating a positive effect on cognitive distortions. However, daily assessments did not show an increase in adolescents’ motivation for behavior change. Positive effects of Street Temptations and virtual reality, as well as areas for improvement, were highlighted in all interviews. Recommendations for further development and implementation are discussed. Overall, this study provides preliminary support for the use of Street Temptations as an add-on intervention in forensic youth care.

**Supplementary Information:**

The online version contains supplementary material available at 10.1007/s10802-025-01313-1.

## Trial registration number

Central Committee on Research Involving Human Subjects NL75545.029.20. Netherlands Trial Register NL9639; https://www.trialregister.nl/trial/9639 (30.07.2021).

**Supplementary Information:**

The online version contains supplementary material available at 10.1007/s10802-025-01313-1.

## Introduction

In the beginning of the 20th century, the *Oxford English Dictionary* introduced the word ‘mentalize’ (Allen et al., 2008). Since then, the construct has grown to be known as a crucial ability to navigate human social life (Gergeley et al., 2002). Mentalization, as a mental process, enables us to interpret behavior of both ourself and others as meaningful, using intentional mental states, e.g. thoughts, feelings, and motives, as a basis for such interpretations (Allen et al., 2008; Bateman & Fonagy, 2004).

Since its first introduction, a substantial amount of work has been focused on discovering how mentalization is related to a variety of mental health problems and how mentalization-based treatment approaches may help to alleviate such problems. Mentalization is operationalized as reflective functioning, which may help to understand the nature of mental health problems that can be related to mentalization. For example, when encountering issues in mentalization, i.e. reflective functioning, adequately interpreting others’ motives and behavior becomes difficult (Baglio et al., 2016; Happé, 1994; Yirmiya et al., 1998). Furthermore, problems regarding self-awareness and -regulation can be expected (Gergeley et al., 2002). As a result, misunderstanding social cues becomes more likely and interpersonal communication can become more impulsive (Möller et al., 2014). This misinterpretation of social information, through disturbance of a social cognitive process like mentalization, may result in aggressive or antisocial behavior (Arsenio, 2010; De Castro & Van Dijk, 2017).

Mentalization may function as both a possible protective factor, as well as a potential risk factor for such disruptive behavior. Consequently, to mitigate this behavior, focusing on the enhancement of mentalization may be a relevant target in therapy (Klein Schaarsberg et al., under review). At the same time, mentalization has been found to function as a prerequisite to engage in and benefit from psychotherapy, also significantly predicting premature treatment dropout (Allen et al., 2008; Jørgensen et al., 2021).

Although still in its infancy, the relevance of mentalization and mentalization-based treatment approaches particularly in relation to youth with (serious) disruptive behavior problems are gaining interest. By means of the current study we hope to instigate this interest, as mentalization presents itself as a promising but also understudied concept in relation to this population (Kasper et al., 2024). More specifically, to explore the added value of a mentalization-based approach to forensic child and youth care, Street Temptations was developed and studied.

### Street Temptations

Street Temptations (ST) is an add-on intervention for adolescents with disruptive behavior problems. Adolescents engage in perspective-taking exercises using fictional and real-life scenarios. They create backstories for the characters involved and reflect on connections between observable behavior and internal mental states. This reflective process is the essence of mentalization, as previously explained (Choi-Kain & Gunderson, 2008; Fonagy et al., 1998).

An innovative strength is the use of virtual reality (VR). First, VR aids perspective-taking exercises by incorporating visual elements into therapy, reducing cognitive demands for creating mental representations of situations that are often used for such exercises (Cornet & van Gelder, 2020; van Gelder et al., 2019). This makes VR especially beneficial for ST. Second, this visual support is likely to benefit (part of) the target population, including adolescents with mild-to-borderline intellectual disabilities, who may require visual aids for effective treatment (de Wit et al., 2023; Moonen & Kaal, 2017; Vermaes et al., 2014). Third, VR may also appeal to a tech-savvy generation (Lochman et al., 2019; Weisz et al., 2018), while providing a safe yet realistic environment to challenge perspective-taking in social situations involving disruptive behavior (Bailenson, 2018). Fourth, adolescents may have difficulty engaging in therapeutic conversations (O’Keeffe et al., 2018; Stige et al., 2021). Using VR could help expedite the conversation between therapist and adolescent, having not only to rely on talking strategies. ST’s VR content, and the rest of the program, have been developed in close collaboration with adolescents and practitioners (Klein Schaarsberg et al., 2023, 2024).

By using mentalization as an important mechanism, ST’s primary aim is to challenge adolescents to reflect on both their own and others’ behavior and underlying mental states. In doing so, focus lies on the reinforcement of adolescents’ treatment responsivity, as not all adolescents with disruptive behavior problems seem to profit from existing evidence-based treatment options (Riise et al., 2021). More specifically, it is aimed to enhance two particular treatment responsivity indicators, being motivation for behavior change and self-serving cognitive distortions.

### Treatment Responsivity Indicators

Motivation for behavior change is an important factor for treatment responsiveness (Miller & Rollnick, 2002; Olver et al., 2011). However, in the case of adolescent disruptive behavior, intrinsic motivation for behavior change can be particularly low (Brauers et al., 2016; Englebrecht et al., 2008; Harder et al., 2012; Hillege et al., 2017). Therefore, addressing motivation appears vital for sustainable behavioral changes and treatment success (Carl et al., 2020; Harder, 2018).

To foster motivation for behavior change, one must recognize problematic outcomes of certain behavior, desire different behavior, and be open to seeking help in this instance (Drieschner et al., 2004; Ward et al., 2004). To achieve this, a certain level of self-reflection and insight is required (te Velde et al., 2015). For that reason, as self-reflection and insight are part of mentalization, ST is assumed to be of added value for the enhancement of adolescents’ motivation. Through exercises that challenge adolescents to explicitly consider mental states while attributing meaning to certain behavior, it is attempted to create space for the evaluation of one’s own interpretations and behavior. In that way, ST intends to challenge adolescents to identify problematic outcomes of their own interpretations and behavior, for example, fostering the motivation to change this behavior.

Similarly, such exercises are assumed to be of added value in addressing cognitive distortions. Such self-serving distortions are described as inaccuracies or biases in relation to thoughts, for example, which are used to attribute meaning to behavior. An example is a tendency to interpret ambiguous behavior of someone as hostile, potentially leading to a hostile, inadequate reaction in return (Barriga & Gibbs, 1996; Barriga et al., 2000, 2001, 2008). A negative association between cognitive distortions and treatment response for treatment of aggressive behavior has been found (Smeijers et al., 2018), stating the importance of addressing cognitive distortions in addition to motivation, for instance. It is expected that ST, through taking on others’ perspectives and actively attributing meaning to behavior, will help to challenge possible inaccuracies and biases in this process of attributing meaning as well (Gibbs et al., 2013). In other words, during the ST exercises therapists can help adolescents to see a different perspective, for example, and to identify other interpretations of certain behavior.

### Potential Treatment Outcome

Advancing on the expectation explained in the previous paragraph, it may be relevant to take emotion regulation strategies into account as a preliminary treatment outcome of participating in ST. Negative distortions, like the ones discussed, have been found to positively correlate with usage of non-adaptive regulation strategies, for example (Deperrois & Combalbert, 2022). Furthermore, emotion regulation is assumed to be highly relevant in relation to disruptive behavior (Njardvik et al., 2022; te Brinke et al., 2021). Although ST’s focus is not on teaching different emotion regulation skills per se, its primary focus on advancing reflection and perspective taking could translate into improvement of one specific emotion regulation strategy: “reappraisal.” This strategy applies to the meaning or self-relevance that is attributed to a certain situation (Gross, 1998, 2015). Therefore, it was deemed relevant to discover how ST’s focus on reflection and perspective taking, and the application thereof through the regulation strategy “reappraisal”, related to a participant’s perceived control over (impulsive) behavior in moments of high arousal.

### The Current Study

The purpose of the present study was to provide a first and thorough exploration of ST’s added value as an innovative VR-based add-on intervention for youth with (serious) disruptive behavior problems. The main focus was on the effects of a mentalization-based approach on motivation for behavior change and cognitive distortions, as important treatment responsivity indicators, as well as on additional effects on emotion regulation as a potential treatment outcome. These effects were studied by applying a single-case experimental design (SCED) in two research settings, combined with additional quantitative measures as well as a qualitative evaluation of the intervention and the VR component more specifically.

A SCED study, involving repeated measurements on individual subjects before, during, and after participating in an intervention, allows individuals to serve as their own controls (Krasny-Pacini & Evans, 2018). Consequently, a SCED study ideally fits clinical research involving small and heterogeneous populations that are difficult to study using standard group designs like the randomized controlled trial. The population targeted by ST fits this description. Additionally, extensively studying a few participants enhances the understanding of individual cases and enables the discovery of individual intervention effects amidst performance variability (Normand, 2016). Altogether, these characteristics ensure that a SCED seems especially suited for this current study. By conducting our SCED study, we aimed to provide valuable insights into ST as an add-on intervention and contribute to the advancement of forensic youth care.

## Methods

The protocol for this study has been published and will be referenced for additional information where appropriate (Klein Schaarsberg et al., 2022). We followed the Single-Case Reporting Guideline in BEhavioural Interventions (SCRIBE) 2016 for the reporting of our single case study (Tate, Perdices, Rosenkoetter, Shadish, Tate et al., 2016; Tate, Perdices, Rosenkoetter, Shadish et al., 2016).

### Design

**Fig. 1:**
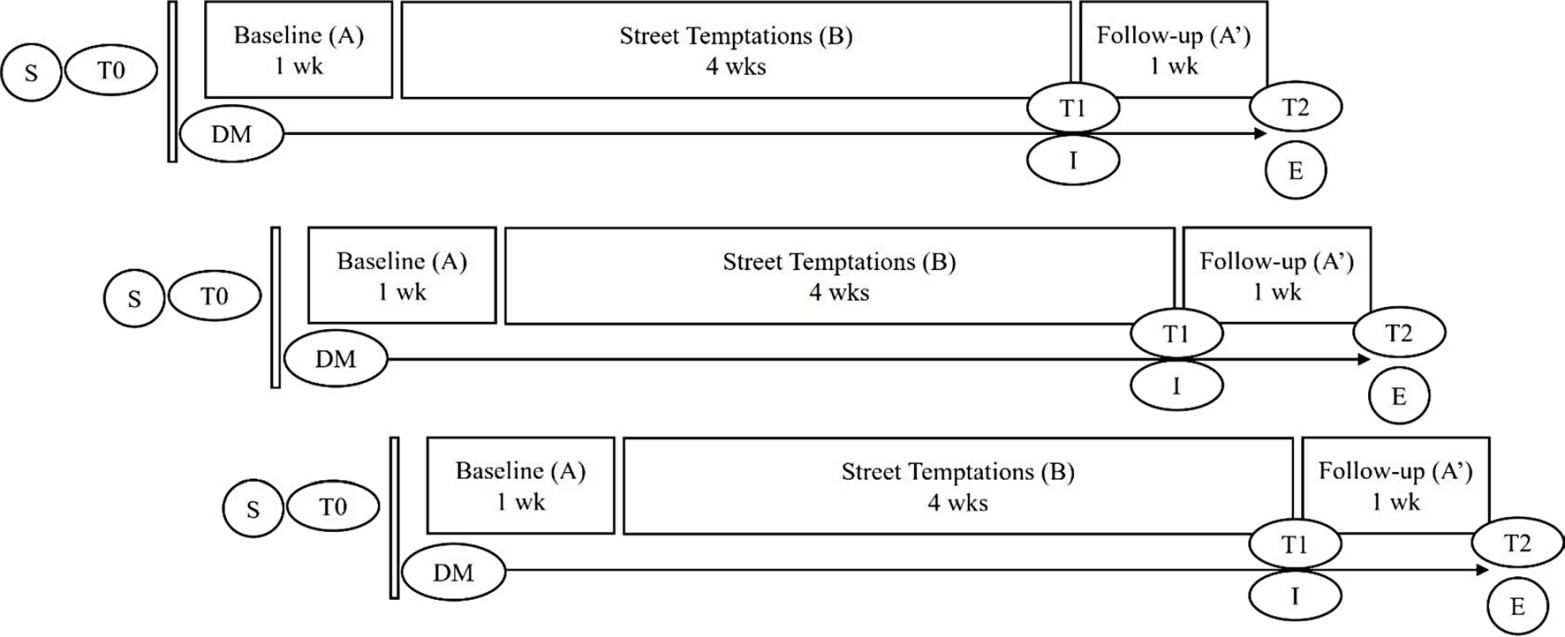
Overview of the study design for three cases. Note. The daily repeated measurements start directly after T0, on the same day; DM = daily repeated measurements; E = end of daily measurements; I = individual interviews with adolescent and ST therapist; T0 = pretreatment assessment; T1 = posttreatment assessment; T2 = follow-up assessment; wk = week

A non-randomized, non-concurrent, multiple-baseline design with replication across single participants was applied (see Fig. 1). Randomization across different baseline lengths was not performed, as baseline lengths would inherently vary due to natural circumstances in clinical practice. Moreover, mandatory waiting would most likely increase the already substantial risk of dropout within this population. Additionally, baselines would naturally be non-concurrent because it was impossible to assign all participants to a therapist at the same time, resulting in varying time lapses between phases across participants in any case. Blinding of participants, ST therapists, and researchers to any component of the study was not used, because this was not feasible due to the nature of the study.

Participants were asked to complete daily repeated measurements during a baseline phase (phase A), an intervention phase (phase B), and a post intervention follow-up phase (phase A’). The baseline phase functioned as a control and was therefore compared with the intervention and follow-up phases. The baseline phase lasted a minimum of 7 days, to keep the baseline length as short as possible to limit the burden on participants while still getting a representative estimate of the outcomes at baseline. The exact start date of the intervention depended on the participants’ presence and the ST therapists’ schedules. The intervention phase lasted at least four weeks, accommodating two ST sessions of 45–60 min per week. When this pace was not achieved, for example, because participants did not attend appointments, the intervention phase was extended. After completing all ST sessions, participants entered the follow-up phase, lasting a final 7 days.

Secondary outcomes were assessed by means of pre-, post-, and follow-up measurements. In addition, separate interviews with participants and ST therapists were performed at the end of the intervention phase. Lastly, 3 and 6 months after ending the study period, participants were approached again for an interview, to evaluate potential long-term effects.

### Procedural Changes

Certain amendments to the original protocol have been made before and during the current study. These amendments followed both from practicalities as from our evolving understanding of the research design in combination with clinical practice. For a detailed description of the amendments, please see Supplementary Material 1.

### Selection Criteria

Adolescents had to meet the following criteria to be eligible for inclusion, assessed by means of clinical judgment: (1) aged between 12 and 18 years, (2) antisocial or externalizing behavioral problems, (3) deficits regarding cognitive distortions or treatment motivation, (4) presence or risk of delinquent behavior, (5) assigned to ST after multidisciplinary consultation, (6) expected stay of at least 2 months, and (7) basic understanding of mobile apps. Clinical judgment, rather than standardized assessment, was chosen to reduce the burden on adolescents and clinicians. Additionally, creating a homogeneous sample—often achievable through standardized assessments—was not a goal of this study. Instead, we deliberately employed a SCED to accommodate the inherent heterogeneity of the population studied. A potential participant who met any of the following exclusion criteria was excluded from participation: (1) severe physical impairment such as deafness or blindness, (2) severe psychiatric problems such as psychosis or high risk of suicide requiring immediate intervention, (3) trauma from serious violence, (4) epilepsy or serious problems regarding motion sickness, and (5) insufficient understanding of the spoken and written Dutch language.

All ST therapists were extensively informed about the study procedures and the in- and exclusion criteria for participants. When an adolescent was referred to ST and was thought to be eligible to participate in the study based on pre-screening, ST therapists informed the adolescent about the study. In case the adolescent was interested and gave oral permission to be approached, an informed consent appointment was planned. Written informed consent was signed when the adolescent agreed to participate in the study. In the case of a minor (12–16 years), parents, or a legal guardian, had to sign a written informed consent as well. Adolescents who did not agree to participate in the study, did not start with ST and received care as usual.

### Setting

The participants were recruited across two different settings. The first setting entailed a rural (secured) residential youth care facility in the province of South Holland, the Netherlands (residential treatment setting). This location also included a school providing secondary special education, partly connected to the residential care facilities. Participants were recruited from both the (secured) living groups as well as from the school. The other setting entailed a specific department of a rural youth care provider in the province of North Holland, the Netherlands (ambulatory treatment setting). This department provides resocialization trajectories for adolescents with severe behavioral problems who dropped out of school, for example. The populations of both settings are characterized by multiple and complex externalizing behavioral problems, sometimes in combination with internalizing problems. Psychiatric and addiction problems can exist as well. The adolescents often come from unstable and problematic family situations and delinquent behavior can be present.

### Participant Characteristics

Within the first setting (residential treatment), 14 adolescents have been screened between September 2021 and October 2023. Of these 14 adolescents, 7 were not approached for study participation due to a negative screening outcome, or because the organization decided that execution of the study was no longer feasible for their organization. Of the other 7 adolescents that were approached, 4 adolescents signed informed consent to participate in the study, of which 3 were included and 1 was not included because parents did not agree with participation of their child.

Participant 1 dropped out during the baseline phase. The intervention could not start because of covid quarantine, and in the meantime the problems of the participant worsened making other care more appropriate. Participant 2 dropped out between inclusion and pre-measurement. He lost his motivation to participate because it took too long to obtain permission from his legal guardian. Participant 3 dropped out during the baseline phase. Her problems worsened throughout the baseline phase, leading to a transfer to another care facility. None of the 3 participants completed measurements in at least two phases, therefore they will not be further discussed.

Within the second setting (ambulatory treatment), 25 adolescents have been screened between November 2022 and December 2023. Of these 25 adolescents, 12 were not approached for study participation due to a negative screening outcome, practical reasons, or because study inclusion had terminated in the meantime. Of the other 13 adolescents that were approached, 5 adolescents signed informed consent to participate in the study, and all 5 were included.

Participant 1 dropped out during the intervention phase. He disappeared for unknown reasons and upon return he was not motivated anymore to continue participation. Participant 2 completed the entire study period. Participant 3 dropped out during the intervention phase, because his motivation declined as it was not allowed that the time spent for ST and the study would shorten his penalty. Participant 4 dropped out during the intervention phase as well because another treatment focus became more important. Participant 5 completed the entire study period. Participants 3 and 4 did not complete measurements in two phases or more, therefore they will not be further discussed. Participants 1, 2, and 5 completed measurements in at least two phases, meaning that these participants will be further discussed.

### Ethics

This study was conducted in accordance with the principles of the World Medical Association Declaration of Helsinki (World Medical Association, 2013) and in accordance with the Medical Research Involving Human Subjects act. Ethics approval for this study was granted by the independent Medical Ethical Committee of Vrije Universiteit medical center (reference number: 2021.0114).

### Measures

#### Daily Repeated Measures

The daily repeated measures were assessed by means of a 14-item questionnaire, constructed by the authors. We assessed cognitive distortions (3 items), treatment motivation (5 items), and emotion regulation (2 items). These constructs were assessed once a day at the end of the day, randomly ordered, in the format of a digital self-report questionnaire for the adolescents. The items were based on the How I Think questionnaire (HIT; Nas et al., 2008), the Adolescent Treatment Motivation Questionnaire (ATMQ; Van der Helm et al., 2013), the Regulation of Emotion Systems Survey (De France & Hollenstein, 2017; Medland et al., 2020), the Ecological Momentary Assessment (EMA) item repository (Kirtley et al., 2019) and the Difficulties in Emotion Regulation Scale (DERS; Gratz & Roemer, 2004). The questionnaire was administered through the m-Path app (Mestdagh et al., 2023), which was installed on the mobile phone of the respective adolescent during pre-measurement. Data resulting from the m-Path app was transferred to a Castor Electronic Data Capture (Castor EDC) database (van Linschoten, 2023).

Responses to each item were recorded using a visual analogue scale (VAS). Unlike a Likert scale, which offers fixed response options, a VAS typically consists of a horizontal line representing a continuum between minimum and maximum outcomes. Respondents mark the point that reflects their perceived position along this continuum. The VAS has been shown to be a reliable, valid, and easy-to-administer measure across several research fields (Brazier & Ratcliffe, 2017; Miller et al., 2003). We chose the VAS over the Likert scales from the original questionnaires to offer participants greater flexibility in answering the daily questions. For statistical purposes, the scale ranged from 0 (minimum) to 100 (maximum). The number on the scale was not visible to participants. While the reliability and validity of adapting the respective items from a Likert scale to a VAS in our study have not yet been fully established, this approach builds on evidence supporting the robustness of VAS measures in similar contexts (Arts et al., 2024; Rhee et al., 2017).

Other items of the daily questionnaire assessed how a participant was feeling, and whether they had a ST session that day. The questionnaire ended with an open question so participants could communicate something else if they wanted. The questionnaire was checked with two adolescents from one of the study sites (who would not participate in the SCED study), to receive feedback on the time needed to fill in the questionnaire, the questions, and the idea of daily assessment. Based on the feedback received, no alterations were made. In Supplementary Material 1 we elaborate on the construction process of the questionnaire. The complete daily questionnaire, including the response scales and original questions if applicable, can be requested from the authors.

#### Pre-, Post-, and Follow-up Measures

During pre-, post-, and follow-up assessment, the following constructs were assessed: cognitive distortions, treatment motivation, mentalization (operationalized as reflective functioning) and perspective-taking. We used the HIT (Nas et al., 2008), the ATMQ (Van der Helm et al., 2013), the Reflective Functioning Questionnaire for Youths (RFQY; Ha et al., 2013), the Self-Reflection and Insight Scale for Youth (SRIS-Y; Sauter et al., 2010) and the Perspective Taking (PT) subscale of the Interpersonal Reactivity Index (IRI; Davis, 1980, 1983).

The measurements were assessed through direct data-entry in a Castor EDC database (van Linschoten, 2023). Additionally, after finishing the intervention phase, interviews with adolescents and ST therapists were conducted to explore the overall experiences regarding ST and VR. The interviews were recorded using professional recording equipment. Personal communication throughout each participant’s study period, both with each participant and the ST therapist, served to gather additional information. A comprehensive description of the instruments used can be found in Supplementary Material 1.

### Intervention

**Fig. 2:**
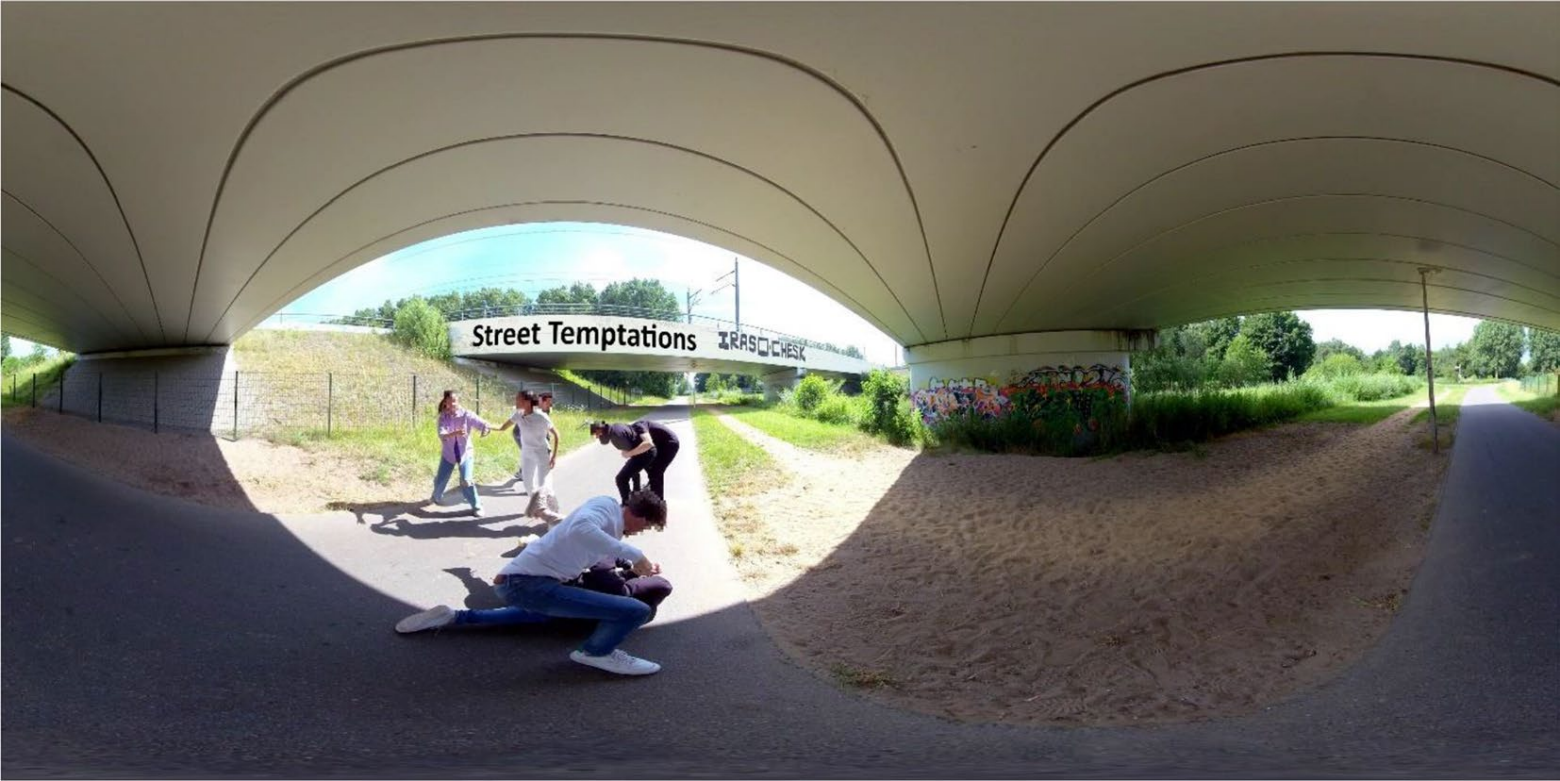
Screen capture VR video ST

ST consisted of seven sessions that were divided over two modules. Each session lasted between 45 and 60 min and was executed individually. The first session served as an intake, the last session as a closing session. In module 1, several perspective-taking exercises were done, which were based on a 360° VR video (see Fig. 2 for a screen capture). By means of this video, youth were presented with a fictional situation depicting (violent) disruptive behavior. A detailed description of the VR video was presented in our recently published paper (Klein Schaarsberg et al., 2024). Several characters played a role in the story and each of the three sessions centered around one of the characters. The exercises were guided by the therapists, whilst providing room for adolescents to take charge by letting them choose different aspects such as which character is discussed when.

Individual backstories were created for each character, using several building block cards that focused on, for example, family, school, and living situation. These cards presented different questions to help adolescents to create a personal story behind the chosen character, such as “What kind of neighborhood does [character] live in?”, “What does [character] spend the most money on?”, “Is [character] a family person?”, or “With whom does [character] call or text the most?”. The therapists let the adolescents choose different cards and then they guided the adolescents in creating the backstory by asking the questions and providing further prompts. When the backstory had been created, adolescents took on the perspective of the respective character and reflected on their experience of the situation. They were explicitly encouraged to connect visible behavior to inner mental states such as thoughts and emotions, emerging from the created backstory. Following, adolescents were asked to reflect on similarities and differences between the character and themselves regarding the provided situation.

In module 2, adolescents chose a personal experience as a basis for the same perspective-taking exercises. They visualized their experience using a VR street view app (Wander, Parkline Interactive, 2019). This app visually transported adolescents to the exact location where their experience had occurred. Using this visual cue, the adolescents described the situation aloud. While they were immersed in the visualization, the VR glasses streamed to an additional screen, allowing the therapist to observe in real-time. The therapist could also ask questions to support the visualization, for example. For further details regarding ST, we refer to the protocol and the description of ST’s development (Klein Schaarsberg et al., 2022, 2023). For additional information about the equipment used and how procedural fidelity was monitored, please see Supplementary Material 1.

### Analysis

All data was summarized at individual participant level. For the daily repeated measurements, data was visualized graphically using the shiny app Single Case Design website (Bouwmeester, 2021) and inspected accordingly. For this inspection, we deliberately chose a descriptive approach rather than to follow available SCED guidelines for visual analysis such as reported by Lane and Gast (2014) including quantification of change in trend, level, and stability of data assessed within and between conditions. Both the nature and conduct of our study did not lend themselves adequately to the application of these guidelines, as the data contained many missing values, intrapersonal outliers, and fluctuations. Therefore, we decided to describe only the visible patterns and limit quantification of the data. Qualitative outcomes were approached alike and described per participant, to illustrate experiences and processes.

Data collected at pre-, post- and follow-up assessment were analyzed by means of a Reliable Change Index (RCI; Jacobson & Truax, 1991). When the RCI exceeded (-)1.96 it was assumed that a statistically reliable change had occurred between two phases, using a 0.05 significance level (Jacobson & Truax, 1991). Because the HIT provided a standardization that classifies scores as non-clinical, borderline clinical or clinical, we chose to use this standardization to indicate clinical significance rather than to focus on an RCI. No clear norms were available for the remaining questionnaires, so we calculated RCIs for those outcomes. A detailed description of how the RCIs were calculated can be found in Supplementary Material 1.

## Results

To avoid information overload in the paper itself, we have chosen to provide a summary of the results of the three participants in the main text. We will highlight a number of results per participant, complemented by an overarching synthesis of the results found across the three participants. Elaborative descriptions about participants’ backgrounds and their study trajectories are given in Supplementary Material 2. Readers interested in a comprehensive exposition of all results can find so in Supplementary Materials 3 (Jason), 4 (Steve) and 5 (Aron). The names that are used to describe the participants are fictitious. Demographic information can be found below, in Table 1.

**Table 1:** Participant demographic information

Participant	Jason	Steve	Aron
Sex	Male	Male	Male
Age	16	16	17
Country of birth	The Netherlands	The Netherlands	The Netherlands
Country of birth mother	African country	South American country	European country
Country of birth father	African country	Unknown	Unknown
First language	Dutch	Dutch	Dutch
Highest education completed	Vocational education	Primary education	Primary education
Current education	Secondary vocational education, level 1 of 4	Vocational education	Secondary vocational education, level 2 of 4
Living situation	Living at home, with mother and younger sister.	Living at home, with mother and older sister.	Living out-of-home, at a care facility in Amsterdam.
Parental marital status	Divorced	Divorced	Unmarried
Contact with the police and justice system	Yes	Yes	Yes

### Jason

Jason was a 16-year-old boy, included in the study on January 10, 2023. Jason was proposed to participate in ST because of violent delinquent behavior, both as victim (for which he started trauma therapy) and as offender. There were suspicions of cognitive distortions that might have played a role in his delinquent behavior, particularly in his last offence. He appeared to be motivated for treatment.

#### Daily Measurements

In total, Jason participated in the study for 74 days, from the first completed measurement until the last (21 days baseline, 53 days intervention, 0 days follow-up). The ST sessions were executed on day 22 and 30. Jason completed 22 daily questionnaires, of which 14 during baseline (7 missings) and 8 during intervention (45 missings).

**Fig. 3:**
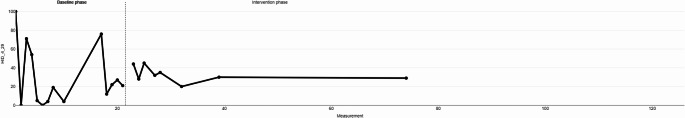
Item 4. People tried to bother me today – totally disagree (0) *→* totally agree (100)

Regarding cognitive distortions it was expected that scores would decrease throughout the study period. The baseline phase of the three items showed more variation in scores than those in the intervention phase, which followed a more stable course. Item 4 (Fig. 3), assessing whether Jason perceived other people to try and purposefully bother him that day, showed the most favorable trend. Although the lowest scores from the baseline did not recur during the intervention, the high peaks did not recur either. Therefore, comparing baseline to intervention, it seemed that Jason both did and did not interpret other people as trying to get in his way during baseline, whereas he less interpreted other people as such during the intervention phase.

**Fig. 4:**
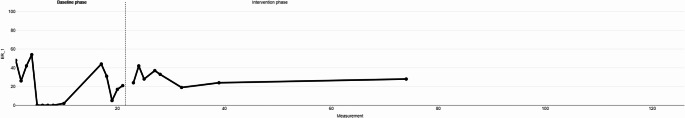
Item 10. In response to my emotions today, I looked at things from a different angle – not true (0) *→* true (100)

**Fig. 5:**
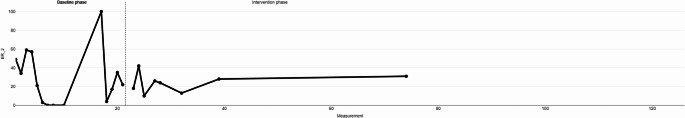
Item 11. When I am upset, I lose control over my behavior – not true (0) *→* true (100)

Considering emotion regulation, illustrated by items 10 and 11 (Figs. 4 and 5), we expected scores to increase for item 10 and to decrease for item 11. Both items showed a positive trend, with a more stable pattern during the intervention phase. For item 10, the lowest scores did not return in the intervention phase, but neither did the highest scores. The scores indicated that, during the intervention phase, Jason more often looked at things from a different perspective than during the baseline phase. For item 11, it appeared Jason perceived to have more control over his behavior when he would get upset throughout the intervention phase, also towards dropping out. Again, for a comprehensive overview of the outcomes, please see Supplementary Material 3.

#### Pre-, Post-, and Follow-up Assessment

For Jason, only pre-measurement outcomes were available meaning no comparison could be made. Regarding the HIT, most scores could be classified within the borderline clinical or clinical range, indicating serious problems on several domains.

On the ATMQ, Jason scored above the 75th percentile at baseline, with a score of 2,82, indicating high motivation. His total score on the RFQY was 8,74, out of a maximum score of 12. Regarding the SRIS-Y, Jason’s score on the Self-Reflection skills subscale was 52 out of 66, and his score on the Insight subscale was 25 out of 36. The score of 14 on the perspective-taking scale of the IRI indicates below average perspective-taking skills.

#### Qualitative Data

Because Jason dropped out of the study during the intervention phase, no interview could be conducted. Therefore, no qualitative data were available.

### Steve

Steve was a 16-year-old boy, included on February 13, 2023. Steve was proposed to participate in ST because of substantial problems with perception and interpretation, related to his violent behavior. He appeared to be motivated for participation.

#### Daily Measurements

In total, Steve participated in the study for 73 days, from the first completed measurement until the last day of the follow-up phase (9 days baseline, 55 days intervention, 9 days follow-up). The ST sessions were executed on day 10, 24, 30, 37, 45, 52, and 64. Steve completed 32 daily questionnaires, of which 9 during baseline (0 missings), 18 during intervention (37 missings), and 5 during follow-up (4 missings).

**Fig. 6:**
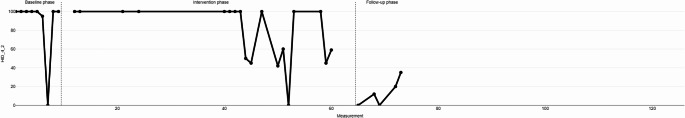
Item 2. If I lose my temper, there is nothing I can do about it – totally disagree (0) → totally agree (100)

**Fig. 7:**
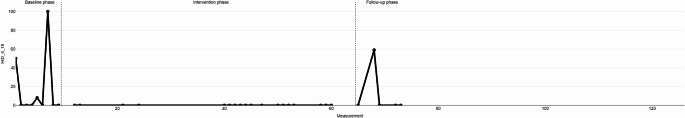
Item 3. No matter how hard I try, I can’t help getting in trouble today – totally disagree (0) → totally agree (100)

For cognitive distortions, it was expected that scores would decrease. Especially item 2 (Fig. 6) showed an interesting pattern. Apart from one outlier in the baseline phase, the scores were all as high as possible throughout the first half of the intervention phase, indicating that Steve totally agreed with the statement. Then, the scores started to fluctuate, exactly when Steve transitioned from module 1 to 2 and thus started to discuss his personal experience; the last session of module 1 was on day 38, the first of module 2 on 45. Steve started to agree less with the statement that there is nothing he can do about losing his temper, and this trend continued in the follow-up phase. In this last phase no high scores were reported anymore. Regarding item 3 (Fig. 7) the intervention phase only showed totally disagreeing scores. This was a positive contrast with the baseline and follow-up phases, which did show agreeing scores.

**Fig. 8:**
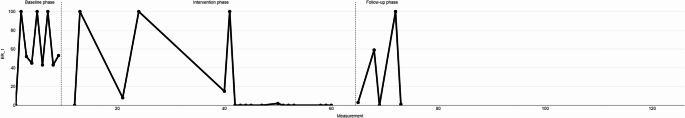
Item 10. In response to my emotions today, I looked at things from a different angle – not true (0) → true (100)

**Fig. 9:**
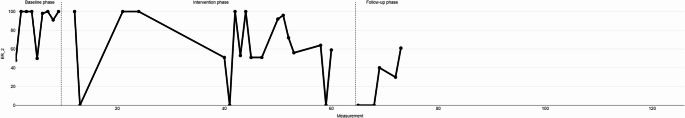
Item 11. When I am upset, I lose control over my behavior – not true (0) → true (100)

The outcomes for the two emotion regulation items showed considerable spread and fluctuation. We expected scores to increase for item 10 (Fig. 8) and to decrease for item 11 (Fig. 9). For both items, a similar pattern could be observed, with deviating or fluctuating scores in the second half of the intervention phase when Steve’s personal experience was discussed. Again, the confrontation of discussing his personal experience may have played a role in the patterns observed. Regarding item 11 specifically, Steve appeared to perceive more control over his behavior when he would get upset at this point. This seemed to be carried over into the follow-up phase. Again, for a comprehensive overview of the outcomes, please see Supplementary Material 4.

#### Pre-, Post-, and Follow-up Assessment

For Steve, pre-, post-, and follow-up measurements were available. The tables displaying all scores and RCIs are presented in Supplementary Material 4. The outcomes are summarized below.

Almost all HIT-scores fell within the non-clinical range, indicating little problems regarding cognitive distortions. However, for the subscale Blaming Others, the score deteriorated from pre- to post-measurement, falling in the clinical range at post-measurement. A week later the score had improved a little, falling within the borderline-clinical range. The scores for Physical Aggression and Stealing had deteriorated into the borderline clinical range at post-treatment, having improved again into the non-clinical range a week later at follow-up. The score for Lying improved from pre- to post-treatment.

The changes in Steve’s scores on the ATMQ were statistically unreliable according to the computed RCIs, indicating no reliable change in motivation for behavior change. Based on the computed RCIs for Steve’s scores on the RFQY no reliable change in reflective functioning could be noted as well. The changes in Steve’s scores on the Self-Reflection subscale of the SRIS-Y were statistically reliable, suggesting a reliable increase in self-reflection. The computed RCIs for Steve’s scores on the Insight subscale of the SRIS-Y indicated that both the increase and decrease in insight from pre- to post-measurement and from post-measurement to follow-up respectively were statistically reliable. Lastly, Steve reported increasing scores on the PT-IRI. According to the computed RCIs the increase from pre- to post-measurement was statistically reliable, suggesting an increase in perspective-taking tendencies.

#### Qualitative Data

Both Steve and his ST therapist participated in the interviews at post-measurement. Both mentioned they enjoyed doing ST. Steve said that his participation has primarily helped him to stand in someone else’s shoes. This matched with his increased scores on the PT-IRI. Switching perspectives was also what interested his ST therapist the most. Furthermore, Steve stated that ST has helped him to think but also talk more about his own thoughts and feelings, both during treatment but also at home:

Because now, when I do not feel good for example, I think about whether something has happened and before, I thought ‘I’m just like that’, so it did help me to think about my feelings.… Yeah, I can just talk about it more, at first, I just thought ‘no I don’t want to talk about it’, but now I just open myself up more because I notice it helps me to be more open.… That is really important.

In turn, this contributed to feeling happier, which is also seen in item 1 of the daily questionnaire. However, the ST therapist mentioned that Steve had also done Eye Movement and Desensitization and Reprocessing (EMDR) therapy before ST, and she thought that this probably largely contributed to the improvements in his mood.

The added value of VR was mainly that it gave a more realistic touch to the exercises. Both Steve and his ST therapist were impressed with the effect the street view component had, which was used to discuss a personal experience from Steve. The ST therapist explained:

I obviously knew the reason this boy is coming to us [ambulatory treatment setting], of course I knew that situation very well, but I had never actually seen it before, and I thought that was really of added value.… He could completely explain ‘yeah that’s where the neighbor came from and there was this and there was that’ so that was really nice.… I also almost had the idea that he was like ‘oh I’m really back at that place’, he also found it kind of thrilling and he also indicated that he had never been back there before.

Furthermore, directly after the first session in which they used the street view component, the ST therapist called the researcher about the session. She said that Steve, by taking on the perspective of the neighbor as a bystander, realized that he had never apologized to this bystander. He stated that perhaps he should still do so because it probably must have been very intense for this person:

He said to me: Shouldn’t we arrange for some flowers or something like that for that neighbor, or can’t we do that anymore after 3 years?.

She was extremely impressed by this realization.

Regarding the VR video, it was mentioned that it could be improved by creating a more intense and streetwise scenario. The current version was a bit too mellow, in relation to what most youths are used to.

### Aron

Aron was a 17-year-old boy, included on October 12, 2023. He was proposed to participate in ST because of having trouble empathizing with others and talking mainly from his own point of view. He showed difficulty assessing how things are for someone else, making the perspective changing exercises particularly relevant.

#### Daily Measurements

In total, Aron participated in the study for 124 days, from the first completed measurement until the last (17 days baseline, 99 days intervention, 8 days follow-up). Aron completed 20 daily questionnaires, of which 13 during baseline (4 missings), 7 during intervention (92 missings), and 0 during follow-up (8 missings).

**Fig. 10:**
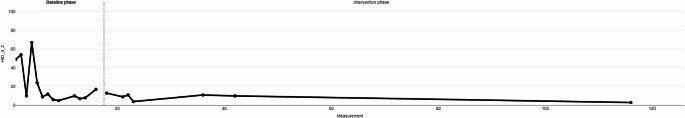
Item 2. If I lose my temper, there is nothing I can do about it – totally disagree (0) → totally agree (100)

**Fig. 11:**
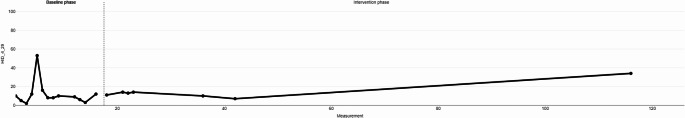
Item 4. People tried to bother me today – totally disagree (0) → totally agree (100)

Regarding cognitive distortions, we expected a decline in scores throughout the study period. Items 2 (Figs. 10) and 4 (Fig. 11) showed some variability in the beginning, and already during the baseline phase scores declined. They stayed low during the intervention phase, apart from item 4’s last measurement. The scores indicated that Aron increasingly thought that he could do something about losing his temper, and that he less interpreted other people as bothering him.

**Fig. 12:**
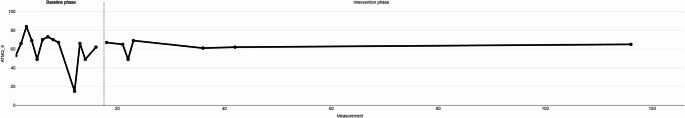
Item 8. I thought about my behavior today – not true (0) → true (100)

The scores for motivation (items 5 through 9) were expected to increase throughout the study period. One item, number 8 (Fig. 12), showed this expected pattern, indicating that Aron started to think more about his behavior throughout his participation in ST.

**Fig. 13:**
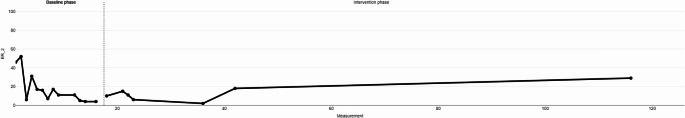
Item 11. When I am upset, I lose control over my behavior – not true (0) → true (100)

Looking at emotion regulation, item 11 (Fig. 13) showed a cautiously positive pattern, suggesting Aron perceived to have more control over his behavior when he would get upset. However, it remains unclear to what extent this can be attributed to the intervention, since the change was already observable during the baseline phase. Furthermore, in the second half of the intervention phase scores started to slightly deteriorate again. Again, for a comprehensive overview of the outcomes, please see Supplementary Material 5.

#### Pre-, Post-, and Follow-up Assessment

For Aron, pre-, post-, and follow-up measurements were available. The tables displaying all scores and RCIs are presented in Supplementary Material 5. The outcomes are summarized below.

All HIT-scores fell within the non-clinical range, apart from the score for Assuming the worst (borderline clinical range), indicating little to no problems regarding cognitive distortions. At post-measurement and follow-up all scores fell within the non-clinical range, indicating no problems.

Aron’s scores on the ATMQ increased from pre-measurement to follow-up and the RCI indicated that this increase was statistically reliable. This suggests a reliable increase in motivation for behavior change. Based on the computed RCIs for Aron’s scores on the RFQY no reliable change in reflective functioning could be noted. The changes in Aron’s scores on the Self-Reflection subscale of the SRIS-Y were statistically reliable, suggesting a reliable decrease in self-reflection. The computed RCIs for Aron’s scores on the Insight subscale of the SRIS-Y indicated that the increase in insight from pre- to post-measurement and from post-measurement to follow-up was statistically reliable. Lastly, Aron reported a decrease in perspective-taking tendencies from pre- to post-measurement, which was statistically reliable based on the RCI. His score increased from post-measurement to follow-up, but this change was not statistically reliable.

#### Qualitative Data

Both Aron and his ST therapist participated in the posttreatment interviews. Aron stated that he only participated because his court-imposed conditions mandated him to come to the treatment facility. He was fine with filling this time with participating in ST, but he said he did not learn anything from it, and he did not notice anything to be different. The only thing that was changed, was the new routine of completing the daily questionnaires. Simply put, he said he did not care that much about it all. However, he also mentioned that he found the perspective-taking exercises quite interesting. Aron’s ST therapist explained that she did recognize this attitude from him. Despite Aron himself not being very disclosing, she could tell that he really enjoyed it. Especially the last sessions, which centered around his personal experiences:

Aron was the same way with me at first, he is not very talkative with strangers at all… when we discussed it, he did say he just really liked it. Because now he could also make it visual, that which he has always told me over the past six months during which we talked about, for example, his offence. Or his neighborhood and what he did there, so he thought it was really fun to show that too using Wander.

Moreover, an important added value of using street view in Aron’s case was that he was able to use the application to visualize a situation in a way that would never have been possible in real life; several location bans prohibited him from going to these places. This could not have been replaced with looking at Google Maps on a smartphone for example, according to Aron’s ST therapist:

I think because he could also see that I could watch along and he could just look with his head where he wanted to look, it is all very fast. But I can also follow that speed right away because I can watch everything live along. So, I think street view on a laptop or on a mobile would have been too difficult.

Concerning the other VR element, the video, Aron only stated that it should be improved, without giving specific ideas about relevant improvements. His ST therapist, on the other hand, explained that she primarily thought the video was not intense enough. Nowadays, violence amongst youths simply not only entails fists, weapons really do also play a role, even if they are only used to threaten.

Lastly, social desirability should be considered in Aron’s case. It is striking, for example, that at the follow-up measurement every subscale score of the HIT questionnaire is the same, coming from the fact that Aron only gave the same answer to all relevant questions, without any nuance. His ST therapist also stated:

I think he can be very socially desirable and knows when to be so and with whom to be so.

This means that, even though we established statistically reliable changes on different outcomes, and the HIT questionnaires showed little to no problems for example, the question arises to what extent these changes and outcomes reflect Aron’s reality.

### Overarching Synthesis

Considering the outcomes of the daily measurements of the three participants collectively, there appeared to be positive changes in cognitive distortions and emotion regulation. However, these findings should be interpreted cautiously as they may be influenced by the study’s limitations. Nonetheless, looking at the items from both constructs, at least one item from each participant showed a positive pattern. Other items showed either no change, or a slightly negative change in pattern. Furthermore, cautiously positive patterns for 2 of the 3 participants in terms of their mood were noted (Jason and Steve). Considering motivation, we could not distill a positive effect of participating in ST. We observed either no change in scores, or a negative change, apart from 1 item from 1 of the participants (Aron).

The pre-, post-, and follow-up assessments of Steve and Aron showed different outcomes. Regarding cognitive distortions, almost all HIT-scores fell within the non-clinical range, indicating little problems. For Steve specifically, some scores had deteriorated at post-assessment, which had improved again at follow-up. On motivation, Steve did not show a statistically reliable change on the ATMQ, whereas Aron showed a statistically reliable increase. This increase was not consistent with his daily measurements. Looking at the RFQY, both participants did not show a statistically reliable change in reflective functioning. Regarding the SRIS-Y, Steve showed a statistically reliable increase in self-reflection. For insight, a statistically reliable increase could be noted from pre to post assessment, followed by a statistically reliable decrease in insight from post to follow-up assessment. Aron showed opposing results, with a statistically reliable decrease on the Self-Reflection subscale and a statistically reliable increase on the Insight subscale. The same was observed for perspective-taking; Steve’s scores on the PT-IRI reflected statistically reliable improvement, whereas Aron’s score indicated a statistically reliable decrease in perspective-taking skills.

Technical issues pertaining to the VR component emerged across the three participants. The main problem was failing attempts of streaming the VR footage to another screen, so a trainer could watch along with a participant. Another issue was stalling of the video when playing it, for example. These issues could not always be fixed on the spot, hindering execution as planned. Therefore, the technological constraints need further development to improve future execution.

## Discussion

The aim of the present study was to explore the added value of ST to forensic youth care. The primary focus was on the use of mentalization as a main treatment mechanism, and how such an approach could help to address cognitive distortions and motivation for behavior change as treatment responsivity indicators. In total, 8 participants were included in the single-case study, of whom 5 participants dropped out. Three participants completed sufficient data for visual inspection of the results. Two of them completed the entire study period. Considering the outcomes of the 3 participants included in the analysis, all 3 trajectories showed different patterns, both in terms of daily assessment and the pre-, post-, and follow-up assessments. Besides these different patterns, the daily measurements of all participants showed at least one item on cognitive distortions and emotion regulation that changed positively throughout the study period. However, on motivation, no positive effect of participating in ST was found. The added value of VR to ST was clearly stated in the interviews, although areas for improvement were unanimously mentioned.

The positive changes that seemed visible on cognitive distortions and emotion regulation could indicate that, for the adolescents who currently participated, using a relatively short and low-threshold training program such as ST may be beneficial to their treatment responsivity, and potentially to certain treatment outcomes as well. This is hopeful, considering the complexities that are involved with treatment in forensic youth care, though the results warrant careful interpretation given the exploratory nature of the study. To some extent, these results are in line with a recent study, in which reductions in hostile attribution bias were found following a short 5-session modification training (Van Bockstaele et al., 2020). Hostile attribution bias has been widely studied as a specific biased way of attributing meaning to social situations and behavior, and can be considered to be an example of cognitive distortions (Barriga et al., 2008; Brugman et al., 2011; Verhoef et al., 2019). It is likely that, through reappraising hostile inferences and replacing them with other, less hostile interpretations, behavioral reactions can become more controlled and less hostile as well (Gagnon & Rochat, 2017).

Less favorable outcomes regarding motivation were found. Positive effects on motivation, at least concerning daily assessment, were little to not observed. In some cases, certain motivation items, such as trusting therapists or the perceived benefit of going to the treatment facility, already started off on a positive note or even showed a ceiling effect. Currently, it is not exactly clear what these positive observations can tell us. Considering the trajectories of Steve and Aron, who completed the intervention, and the fact that they reported their guidance as being useful in combination with reporting at least some trust in their therapists, it may be the case that some form of a trusting relationship between an adolescent and their therapist is a precondition in order to execute ST. However, Jason’s results are not in line with this possibility. He did not complete the intervention but did report to find his guidance useful as well. Moreover, he reported higher scores regarding trusting his therapist than Steve and Aron. Therefore, both the role of as the effects on trust in relation to ST and motivation, for example, remain somewhat unclear. Furthermore, because of the mentalization-based approach, the primary aim of ST was to challenge youngsters to interpret behavior of both themselves and others, by reflecting on underlying factors such as thoughts and emotions. As a result, the wording of the items on motivation that directly or indirectly related to this aim may have been too abstract. The words “behavior” and “problems” in questions such as “I want to change my behavior” and “I talk about my problems” can refer to different behavior and problems. This may have made it more difficult to specify a particular motivation for behavior change and therefore may have complicated answering the questions. Moreover, it is possible that these generally formulated questions led to a more trait-like rather than state-like assessment, limiting meaningful outcomes on a daily basis (Myin-Germeys & Kuppens, 2022).

A common pattern that was seen in the daily measurements is that the variability in the results seemed to decrease as time passed. It is not exactly clear what this indicates. It could be that these patterns are the result of a time-bound pattern seen more often in repeated assessment, especially in repeated subjective assessment. This pattern is known as the initial elevation bias, in which consecutive subjective reports may tend to exhibit higher values during initial assessments. These higher values are followed by a slight decline that eventually stabilizes (Myin-Germeys & Kuppens, 2022; Shrout et al., 2018). It is unclear why this bias occurs, but in any case, it means we should interpret the current findings with express caution.

The initial elevation bias may also play a part in the fact that common SCED guidelines recommend researchers to await baseline stability, before transitioning into the consecutive phase (Kazdin, 2011; Kratochwill et al., 2010). In our study, transitioning from baseline to intervention did not depend on baseline stability. In addition, we did observe some changes in the daily measurements already occurred during baseline (possibly due to an initial elevation bias, as previously explained). As a result, we currently cannot make convincing statements about whether the visible patterns could actually be attributed to participation in ST specifically. The question is, however, to what extent awaiting baseline stability would have reduced this problem. Recently, this recommendation has been questioned due to a lack of empirical evidence to support this recommendation (Lanovaz & Primiani, 2023). Moreover, we should consider that certain concepts fluctuate over time, although, in theory, there are assumed to be static (Myin-Germeys & Kuppens, 2022). Therefore, it seems reasonable to consider the actual relevance of baseline stability in psychologically oriented studies. Perhaps it is of greater importance to obtain a representative snapshot during baseline, which may include realistic fluctuations.

Considering the VR component of ST, being the 360° video and the VR street view application, the qualitative data showed that these relatively simple VR tools added a lot of value to the exercises and yielded promising results. In particular, the video and street view application seemed to foster the conversation between the therapist and the adolescent. Furthermore, it was of specific value for therapists to actually *see* a certain place that an adolescent was talking about, when explaining a situation that had happened there. Even if the therapists already knew the story. Moreover, in one case the use of VR street view even led to a breakthrough in the way he considered the perspective of one of the bystanders. Previous research has shown similar outcomes in terms of how a VR tool may help to facilitate therapeutic communication (Falconer et al., 2019). Findings like these are important, considering the difficulties that can emerge when pursuing adolescents to engage in therapeutic conversations (O’Keeffe et al., 2018; Stige et al., 2021).

More specifically, these qualitative results hold promise for the mentalizing approach that is central to ST. By instigating conversations about adolescents’ personal lives as well as the lives of others, ample options emerge to challenge them to mentalize. Similarly, other, more interactive forms of VR exercises have been found to generate positive outcomes regarding perspective-taking (Chen et al., 2021; van Loon et al., 2018), a crucial element of mentalization (Derks et al., 2019). Considering the relatively simple forms of VR that were used in ST, not only technologically advanced and highly interactive VR tools are worth considering. In this context, the rapid developments that are ongoing in VR technology provide additional ground for curiosity and positivity toward the future. Who knows what else could be accomplished in improving care for the youngsters involved, given the more advanced possibilities that may yet be explored.

In addition to the positive and facilitating aspects of working with VR, several areas for improvement were highlighted in the interviews, including the technical part of VR. Therapists mentioned that they struggled often to connect the VR headset to a second device for streaming purposes, for example. Therapists were not always able to solve these issues by themselves, having to rely on the executive researcher for assistance. Even with technical support, it was not always managed to solve a problem, which led to frustration, for instance. Moreover, this appealed greatly to the patience of both youth and therapists, something that cannot always be mustered. Technical issues have previously been identified as a weakness of working with VR (Nijman et al., 2020). However, in other recent studies looking into VR-based interventions no to only minor technical issues have been reported (Alsem et al., 2021; Arts et al., 2023). Because the technical reliability within ST should be emphasized in the future, it may be beneficial to learn from success stories like these.

A crucial aspect to discuss in relation to our study, is inclusion and dropout. To some extent, inclusion and dropout are well-known challenges in research (Groeneveld et al., 2009). However, we aimed to include a total of 18 adolescents. Ultimately, only two participants completed the entire study period. Moreover, although we still adhered to a minimum of 3, preferably 5, data points per phase as stated by SCED guidelines (Krasny-Pacini & Evans, 2018; Kratochwill et al., 2013), we cannot ignore the high number of missing values. This means that we must be careful when interpreting the results. It also means that generalization of the current results is not possible due to a lack of a sufficient number of direct and systematic replications (Walker & Carr, 2021). Therefore, the current results should only be interpreted as initial effects, related to the specific youth who participated in this study.

An explanation could be that we did not collaborate close enough with adolescents and practitioners while designing the current study. Consequently, our ideas potentially did not fit well enough with the challenges encountered in daily practice. This could apply both to the feasibility of ST as an intervention, as to the feasibility of the study that was designed and conducted under the current circumstances. That is, it could be that the study itself has been a weak link rather than ST as an intervention. Many adolescents “dropped out” before they could even be included in the study. This suggests that aspects related to the preparatory phase of participating in research (e.g., parents or legal guardians of minors were hard to reach for informed consent purposes), as well as the study design itself (e.g., being asked to fill out a daily questionnaire), potentially did not contribute positively to the inclusion of participants. Therefore, it may be premature to conclude that implementing ST in this setting, with these adolescents, is not feasible. We believe further research, possibly with a refined protocol based on the lessons learned from this initial study, is warranted. At the same time, it is important to critically assess the realistic expectations we might have for future research in this area.

Certain practical and logistic issues have been at play as well in relation to dropout. For example, adolescents suddenly left the facility to go to school or because they got arrested (something that was not intended to be influenced by ST), therapists’ schedules abruptly changed due to an emergency or they could not leave the group in order to execute ST with an individual adolescent, or the challenges within the organization itself were such that no time could be devoted to other things. Because these issues were, at least to some extent, inherent to the current research settings, it is important to consider whether these settings are the most fruitful for certain research purposes. In the current study we were able to explore the added value of ST, including VR, within this complex context. However, for certain future research and implementation purposes, such as studying the effect of the VR components specifically, a school context would be worth considering as well, for example (Cummings et al., 2022; Richter et al., 2022).

In any case, we must acknowledge that the problems concerning inclusion and dropout may have created a selection bias. On the other hand, it seems too early to draw any definitive conclusions on whether ST as an intervention is not feasible or acceptable, or that the current study was insufficiently feasible and acceptable to conduct under the current conditions.

### Future Directions

Based on the current study and its outcomes, several implications for future directions can be noted. First, ST, as a VR-based add-on intervention, appears to offer relatively simple options to incorporate mentalization into treatment and in that way potentially help to enhance the treatment responsivity of adolescents. To further enhance outcomes, it may be worthwhile to consider other forms of delivering the program. For example, in line with the significant positive effects seen after brief intensive trauma-focused treatment (De Jongh et al., 2020; Voorendonk et al., 2020), it may be interesting to implement a brief intensive ST program that is delivered within 1 to 2 weeks. Moreover, we can note positive outcomes, even when the program is not completed in its entirety. Therefore, but possibly also in a brief intensive form, ST may be of specific value in the context of the often relatively short placements within the judicial, forensic setting (Buysse et al., 2021). Nevertheless, looking at the practical constraints we encountered, options like these would require considerable organizational efforts.

Second, the addition of VR components to treatment within the context of forensic youth care seems to hold promise and therefore deserves further attention. Using VR, especially the street view component, may hold value in other forms of usage as well. For example, it may be relevant for crime analysis, during which somebody gives a factual description of the events that led to a committed crime, as well as one’s thoughts, feelings, and behavior in relation to the crime. As illustrated by our study, the visual support of using VR street view may add to a reflective exercise like this. Also, VR components like the once discussed may be helpful in preparation of confrontations with possible victims, which is gaining interest by means of restorative justice practices (Rossner & Taylor, 2024; Suzuki, 2023). Considering VR, however, it is important to gain a better understanding of how to develop and use VR in a way that is both ethically responsible and suitable for the adolescents aimed at and the situations they can face. This would include a more extensive evaluation of the VR materials used, also consisting of quantitative assessment methods for example. Youth involved in forensic youth care can be used to quite some violence, unfortunately, which can make a relatively mild scenario like the current one not emotionally engaging enough. Therefore, it was suggested to incorporate weapons like guns or knives, for example. However, this is not something that can be done without question; it is not yet clear where the line lies between an emotionally engaging scenario and one that is irresponsible and possibly even harmful. Violent video games, for example, have been found to negatively impact aggression, other problem behaviors, and normative beliefs about aggression (Greitemeyer, 2022; Shao & Wang, 2019; Wei et al., 2022). There is also evidence against these negative effects available, in adults that is (Kühn et al., 2019), but caution is warranted at the very least. In addition, given the boundless opportunities presented by VR and the blurring of boundaries between virtual and real experiences, there exists a moral imperative in the development of VR experiences (Bailenson, 2018; Brey, 1999; Ford, 2001). Therefore, we must reflect on the question whether we *should* create everything.

Last, for future practice-oriented research it may be particularly beneficial to collaborate with adolescents and practitioners in every step of the research process. By collectively thinking about assessment methods and piloting these methods more extensively, for example, more room can be created to incorporate the language of adolescents themselves instead of primarily relying on therapeutic or academic language. This will likely allow for more idiosyncratically oriented research methods as well. In this way, we can try to get as close as possible to the experiences of the adolescents involved and the ways they express themselves. A good example may be found in the Youth Top Problems measure, for example, a psychometrically sound, client-guided approach to assessment (Weisz et al., 2011).

### Strengths and Limitations

An important strength of this study was the ecological validity. The study was performed under daily, uncontrolled circumstances. While these conditions do create some limitations, as will be discussed below, they also make sure that the outcomes are of direct value to clinical practice. In addition, the results showed that progress and change can be difficult to translate into a set of numbers. Therefore, our study approach, including different data collection methods, moments, and informants, helped improve our understanding of the effects. Furthermore, this triangulation decreased possible deficiencies resulting from only using self-report questionnaires (Thurmond, 2001).

Despite these strengths, several limitations must be considered as well. One of the strengths, the ecological validity, simultaneously created one of the limitations. The nature of the study prohibits attribution of the observed outcomes to either ST or other specific circumstances or therapies, for example. Moreover, we observed some changes in the daily measurements already occurred during baseline (possibly due to an initial elevation bias, as explained in the beginning of the discussion). As a result, we cannot make convincing statements about whether the visible patterns could be attributed to participation in ST. Lastly, while clinical judgment was chosen to reduce the burden on adolescents and clinicians and to accommodate the population’s heterogeneity, this approach limits the generalizability of the findings to other contexts or populations. Future research should consider incorporating standardized assessments to strengthen the evidence base and to enhance comparability across studies.

## Conclusions

Conducting our single-case study within the context of forensic youth care proved complex, but also feasible. Moreover, considering the three different research and treatment trajectories that emerged, this individually oriented research design may hold particular potential for studying this intricate population. The positive changes that appeared to come forward could suggest that the adolescents who currently participated have learned to think more about another person’s perspective over the course of ST. Additionally, they seemed to show positive change in the amount of control they perceived to have over their behavior. This may be beneficial for adolescents’ treatment responsivity. Moreover, these outcomes hold preliminary promise for the use of a mentalization-based approach, such as ST, within the complex context of forensic youth care. However, these results did not seem to necessarily lead to them wanting to work more on their own behavior and problems. Therefore, based on the current outcomes, we cannot conclude that participation in ST was helpful in increasing the adolescents’ motivation for behavior change. Working with VR was deemed advantageous, despite several limiting factors. Taken together, both the future development and study of ST and the VR components are warranted, despite the difficulties of taking the first steps. VR, as a tool within ST, appears to be a valuable resource that can be seen as an addition to the therapeutic toolbox. Furthermore, the positive results that have been highlighted in the current phase of development fuel our curiosity and positivity about future (technological) advancements in forensic child and youth care.

## Electronic Supplementary Material

Below is the link to the electronic supplementary material.

Supplementary Material 1

Supplementary Material 2

Supplementary Material 3

Supplementary Material 4

Supplementary Material 5

## Data Availability

The data that support the findings of this study are not publicly available due to their containing information that could compromise the privacy of research participants.
