# Supplementary material for: The First Step is the Hardest: A Mixed Methods Single-Case Experimental Design Study of a VR-Enhanced Training Program in a Forensic Youth Care Setting
Source: Res Child Adolesc Psychopathol. 2025 Apr 14;53(12):1733–53. doi: 10.1007/s10802-025-01313-1 (PMC12718268; doi:10.1007/s10802-025-01313-1)
Supplement: Supplementary file 1 — Supplementary Material 1 [file 10802_2025_1313_MOESM1_ESM.docx]

Appendix 1 – Methods section

**Procedural changes**

First, while preparing for our study, we concluded that the original VR video and the accompanying visual materials required revision, as the division of roles among the characters increased stereotyping. This was done before continuation of the study, together with adolescents and professionals involved with forensic youth care (Klein Schaarsberg et al., 2024). Consequently, the study has been executed using revised visual materials. Second, one of the research settings was intended to be a school for special education, in addition to the setting of secure residential youth care. However, the school in question decided to stop participating in the study when we improved the visual materials. The research site that replaced this school is described below. Third, randomization across different baseline lengths was no longer considered of value for our particular study, as explained in the Methods section of the main manuscript. Therefore, to limit practical constraints that would be associated with randomization, we decided to discontinue randomization after improving the materials. In line with this, we critically evaluated our daily repeated questionnaire. We decided to keep the daily measurements as concise as possible, resulting in a short, fixed questionnaire as opposed to including one questionnaire as a whole and a complete subscale of another. This also gave room to include two additional items assessing emotion regulation.

**Construction of the daily repeated questionnaire**

*Treatment motivation and cognitive distortions*

When designing the study, no previously published research was available in which treatment motivation or cognitive distortions were assessed on a daily basis. Therefore, the items were based on existing, validated questionnaires. For treatment motivation, the Dutch translation of the Adolescent Treatment Motivation Questionnaire was used (ATMQ; Van der Helm et al., 2013). For cognitive distortions, the Dutch translation of the How I Think questionnaire (HIT; Nas et al., 2008) was chosen.

Both the ATMQ and the HIT were not directly implementable on a daily basis. Therefore, both questionnaires were critically reviewed by the first author, in discussion with the third, fourth and fifth author, to choose a select number of items for daily admission. Review entailed the following:

1. The number of items for daily assessment. Limiting the amount of time spent filling out daily questions was a criterion. Similar items were therefore reduced to one item, as the questionnaire aims to assess consistency over time and not within a moment.
2. The content of the items. ST aims to improve treatment motivation and alter negative attribution bias (e.g., hostile attribution bias). The items should represent this aim. The thought process is central in this aim and actual behavior is less relevant is this instance.
3. The wording of the items. The items should be logical to answer on a daily basis. Therefore, items may be worded differently.

*Emotion regulation*

Emotion regulation has been previously examined using daily repeated measures (Ecological Momentary Assessment; EMA, or Experience Sampling Method; ESM). However, no validated ESM items on emotion regulation currently existed (Mestdagh & Dejonckheere, 2021). Consequently, we chose to base the items on previous studies as much as possible. ST’s exercises primarily focus on advancing reflection and perspective taking, rather than on teaching different emotion regulation skills. Therefore, one specific regulation strategy was assessed, fitting the focus on reflection and perspective taking. This was the strategy “reappraisal”, belonging to the “cognitive change” stage within the process of emotion regulation according to the model of Gross (Gross, 1998, 2015). Several studies have used a single item to repeatedly measure this strategy (Brans et al., 2013; Grommisch et al., 2020; Hiekkaranta et al., 2021; Koval et al., 2022; McMahon & Naragon-Gainey, 2019). The item for the current study was based on these examples, in combination with the reappraisal items of the Regulation of Emotion Systems Survey-EMA (RESS-EMA) questionnaire (De France & Hollenstein, 2017; Medland et al., 2020) and the EMA item repository (Kirtley et al., 2019).

Furthermore, we wanted to discover how ST’s focus on reflection and perspective taking, and the application thereof through the regulation strategy “reappraisal”, related to a participant’s perceived control over (impulsive) behavior in moments of high arousal. For this purpose, 1 item from the Difficulties in Emotion Regulation Scale (DERS; Gratz & Roemer, 2004) was selected, from the subscale “difficulties controlling impulsive behaviors when distressed”. The selected item had the highest factor loading on the subscale, based on several studies (Gratz & Roemer, 2004; Neumann et al., 2010; Victor & Klonsky, 2016). The DERS (both in complete and abbreviated versions), as well as the item selected for the current study, has been the basis for repeated measures on several occasions (Matko et al., 2022; Norman-Nott et al., 2021). Previously, selection of items has also been based on factor loadings (Matko et al., 2022).

**Pre-, post-, and follow-up measures**

*Cognitive distortions*

For the measurement of cognitive distortions, the complete version of the HIT was used. The HIT contains 54 6-point Likert items, that vary from ‘totally agree’ to ‘totally disagree’. Examples of items are “Rules are mostly meant for other people”, and “If I really want to do something, I don’t care if it’s legal or not”. The Dutch version of the HIT showed acceptable reliability and validity (Brugman et al., 2011; Nas et al., 2008). Scores are classified as falling within a non-clinical, borderline clinical or clinical range.

*Motivation for behavior change*

To assess motivation, the complete version of the ATMQ was used (Van der Helm et al., 2017). The ATMQ consists of 11 self-report items with a 3-point Likert scale, ranging from ‘not true’ to ‘true’. An example of an item is “I want to change my behavior together with others”. Reliability and validity of this Dutch questionnaire proved to be good, based on a sample of Dutch adolescents living in a secure residential youth care facility or juvenile justice institution (Van der Helm et al., 2013). A higher score indicates higher motivation, with a score of 1,55 representing the 25^th^ percentile, 2,09 the 50^th^, and 2,44 the 75^th^ percentile. The maximum score is 3.

*Reflective functioning*

Reflective functioning was assessed using the Reflective Functioning Questionnaire for Youths (RFQY; Ha et al., 2013) and the Self-Reflection and Insight Scale for Youth (SRIS-Y; Sauter et al., 2010). The RFQY is a 46-item self-report measure, scored on a 6-point Likert scale ranging from ‘strongly disagree’ to ‘strongly agree’. Examples of items are “People’s thoughts are a secret to me”, “I always know what I feel”, and “I find it difficult to see other people’s points of view”. The questionnaire is adapted from the adult version, the Reflective Functioning Questionnaire (Fonagy et al., 2016), by rewording some items for a better developmental match. Both studies report preliminary support regarding reliability and validity (Fonagy et al., 2016; Ha et al., 2013). Psychometric properties are further supported by a recent Danish validation study (Lund et al., 2023). The RFQY consists of two scales, with a total RFQY score deriving from the sum of both scale scores. Higher scores indicate greater capacity for reflective functioning, with a maximum score of 12 for the total score. Following the analyses of Ha et al. (2013) and Lund et al. (2023), only the total RFQY score was used in the current study. Because no Dutch translation existed at the time of our study, we translated the RFQY in collaboration with the developers and an official interpreter-translator, using a forward-backward translation method. A validation study regarding this Dutch version is currently being conducted.

The SRIS-Y is a 17-item self-report questionnaire, answered with a 6-point Likert scale ranging from ‘strongly disagree’ to ‘strongly agree’. The instrument consists of two subscales, Self-Reflection (SR) and Insight (I), resulting in two separate scores. Examples of items are “I often find it difficult to really understand how I feel about things” and “I usually have a very clear idea about why I have behaved in a certain way”. The original adult version is reported as a reliable and valid measure of self-reflection and insight in adults (Grant et al., 2002). The SRIS-Y appears to be a developmentally-appropriate and psychometrically sound measure of self-reflection and insight in adolescents (Sauter et al., 2010). Higher scores indicate higher self-reflection or insight, with a maximum score of 66 for the SR scale and 36 for the I scale.

*Perspective-taking*

Perspective-taking was assessed using the Perspective Taking (PT) subscale of the Interpersonal Reactivity Index (IRI) (Davis, 1980, 1983). The PT-subscale consists of 7 items, answered on a 5-point Likert scale, such as “I sometimes try to understand my friends better by imagining how things look from their perspective”. The Dutch version of the IRI seems to be a psychometric adequate instrument (de Corte et al., 2007). A higher score indicates higher perspective-taking skills, with a maximum score of 28.

*Demographics*

Socio-demographic information such as age, sex, education level, self-reported ethnic descent, living situation and possible experience with criminal activities was collected using a self-developed demographic questionnaire at pre-treatment. Part of this questionnaire was again used at post- and follow-up treatment, to assess change in criminal activities, for example. Information regarding diagnostic background and treatment history was collected using file information, obtained via the involved ST therapist.

*Street Temptations and VR*

Semi-structured interviews based on the Change Interview (CI) (Elliot et al., 2001) were conducted with adolescents as well as ST therapists to evaluate ST and VR at post-measurement. The purpose of the CI is to obtain information about clients’ understanding about what has changed during therapy, why those changes have occurred, and what factors might have gotten in the way of change. In this way, the interviews enabled learning whether and if so, what changes occurred throughout ST, from both the adolescents’ and ST therapists’ perspectives. Also, these interviews could provide insights in why adolescents and ST therapists thought those changes occurred, and what were potential hindering factors or negative changes. Additionally, for the VR evaluation, participants were asked to reflect on their experience with VR in general and working with the VR material. They were additionally asked to reflect on what VR did or did not add to ST and to reflect on the video used in ST. Additionally they were asked how they thought the VR component could be improved.

**Equipment**

For the VR elements, the Meta Quest 2 headset was used as hardware, together with the ST therapist’s smartphone or laptop for streaming to watch along. The VR video was viewed using a web-based interface, providing a personal link for each participant. The application Wander (Parkline Interactive LLC, 2019) was used for the street view visualization. Furthermore, the playing cards as explained in the Methods section of the main manuscript were used to visually support the exercises. These cards could be put on a whiteboard, for example, to write down the elements of the backstory next to them. ST therapists were provided with an intervention manual explaining the background and execution of the sessions.

**Procedural fidelity**

Procedural fidelity was monitored in various ways. ST therapists had to communicate the date of the first ST session to the executive researcher, in order to correctly administer the transition from baseline to intervention phase. During the intervention phase, ST therapists were asked to fill in session forms using Castor EDC surveys (van Linschoten, 2023). Daily measurements were checked every few days and when measurements were skipped for several days adolescents were contacted to check-in and remind them of the measurements. Additionally, once every to two weeks, participants were tried to call to monitor participant functioning. This was also done through information exchange with ST therapists. They could, for example, inform us when a participant had been arrested. Check-in moments were adjusted to individual participants, so there was no fixed schedule or amount for check-in moments. Lastly, the executive researcher was available for on-the-spot consultation for ST therapists as needed, to answer questions or solve problems directly, in addition to interim supervision outside sessions.

**Analysis – calculation of RCIs**

To calculate the RCIs, we used the following reliability indications and standard deviations. For the ATMQ we used an internal reliability of *α* = .84 and a standard deviation of *sd* = .58 (Van der Helm et al., 2013; Van der Helm et al., 2017). For the RFQY, an internal reliability of *α* = .71 and a standard deviation of *sd* = .92 were used (Ha et al., 2013). The RCIs for the SRIS-Y were calculated using an internal reliability of *α* = .84 for the SR scale and *α* = .77 for the I scale. A pooled standard deviation of *sd* = 1.377 was used for both scales (Sauter et al., 2010). Lastly, for the PT-IRI, we used an internal reliability of *α* = .73 and a standard deviation of *sd* = 4.30 (de Corte et al., 2007).

**References**

Brans, K., Koval, P., Verduyn, P., Lim, Y. L., & Kuppens, P. (2013). The regulation of negative and positive affect in daily life. *Emotion*, *13*. <https://doi.org/10.1037/a0032400>

Brugman, D., Nas, C. N., Van der Velden, F., Barriga, A. Q., Gibbs, J. C., Potter, G. B., & Liau, A. K. (2011). *Hoe Ik Denk Vragenlijst (HID) Handleiding*. Boom test uitgevers.

Davis, M. H. (1980). A multidimensional approach to individual differences in empathy. *JSAS Catalog of Selected Documents in Psychology*, *10*, 85.

Davis, M. H. (1983). Measuring individual differences in empathy: Evidence for a multidimensional approach. *Journal of Personality and Social Psychology*, *44*, 113-126. <https://doi.org/10.1037/0022-3514.44.1.113>

de Corte, K., Buysse, A., Verhofstadt, L. L., Roeyers, H., Ponnet, K., & Davis, M. H. (2007). Measuring empathic tendencies: Reliability and validity of the Dutch version of the Interpersonal Reactivity Index. *Psychologica Belgica*, *47*, 235-260. <https://doi.org/10.5334/pb-47-4-235>

De France, K., & Hollenstein, T. (2017). Assessing emotion regulation repertoires: The Regulation of Emotion Systems Survey. *Personality and Individual Differences*, *119*, 204-215. <https://doi.org/https://doi.org/10.1016/j.paid.2017.07.018>

Elliot, R., Slatick, E., & Urman, M. (2001). Qualitative change prosess research on psychotherapy: Alternative strategies. *Psychological Test and Assessment Modeling*, *43*, 69-111.

Fonagy, P., Luyten, P., Moulton-Perkins, A., Lee, Y.-W., Warren, F., Howard, S., Ghinai, R., Fearon, P., & Lowyck, B. (2016). Development and validation of a self-report measure of mentalizing: The Reflective Functioning Questionnaire. *PLOS ONE*, *11*, e0158678. <https://doi.org/10.1371/journal.pone.0158678>

Grant, A. M., Franklin, J., & Langford, P. (2002). The self-reflection and insight scale: A new measure of private self-consciousness. *Social Behavior and Personality*, *30*(8), 821-835. <https://doi.org/DOI> 10.2224/sbp.2002.30.8.821

Gratz, K. L., & Roemer, L. (2004). Multidimensional assessment of emotion regulation and dysregulation: Development, factor structure, and initial validation of the Difficulties in Emotion Regulation Scale. *Journal of Psychopathology and Behavioral Assessment*, *26*, 41-54.

Grommisch, G., Koval, P., Hinton, J. D. X., Gleeson, J., Hollenstein, T., Kuppens, P., & Lischetzke, T. (2020). Modeling individual differences in emotion regulation repertoire in daily life with multilevel latent profile analysis. *Emotion*, *20*, 1462-1474. <https://doi.org/10.1037/emo0000669>

Gross, J. J. (1998). The Emerging Field of Emotion Regulation: An Integrative Review. *2*(3), 271-299. <https://doi.org/10.1037/1089-2680.2.3.271>

Gross, J. J. (2015). Emotion Regulation: Current Status and Future Prospects. *Psychological Inquiry*, *26*(1), 1-26. <https://doi.org/10.1080/1047840X.2014.940781>

Ha, C., Sharp, C., Ensink, K., Fonagy, P., & Cirino, P. (2013). The measurement of reflective function in adolescents with and without borderline traits. *Journal of Adolescence*, *36*, 1215-1223. <https://doi.org/10.1016/j.adolescence.2013.09.008>

Hiekkaranta, A. P., Kirtley, O. J., Lafit, G., Decoster, J., Derom, C., de Hert, M., Gülöksüz, S., Jacobs, N., Menne-Lothmann, C., Rutten, B. P. F., Thiery, E., van Os, J., van Winkel, R., Wichers, M., & Myin-Germeys, I. (2021). Emotion regulation in response to daily negative and positive events in youth: The role of event intensity and psychopathology. *Behaviour Research and Therapy*, *144*, 103916. <https://doi.org/https://doi.org/10.1016/j.brat.2021.103916>

Kirtley, O. J., Hiekkaranta, A. P., Kunkels, Y. K., Verhoeven, D., Van Nierop, M., & Myin-Germeys, I. (2019). *The experience sampling method (ESM) item repository* <https://doi.org/10.17605/OSF.IO/KG376>

Klein Schaarsberg, R. E., van Dam, L., Widdershoven, G. A. M., Lindauer, R. J. L., & Popma, A. (2024). Ethnic representation within virtual reality: a co-design study in a forensic youth care setting. *BMC Digital Health*, *2*(1), 25. <https://doi.org/10.1186/s44247-024-00081-0>

Koval, P., Kalokerinos, E. K., Greenaway, K. H., Medland, H., Kuppens, P., Nezlek, J. B., Hinton, J. D. X., & Gross, J. J. (2022). Emotion regulation in everyday life: Mapping global self-reports to daily processes. *Emotion*. <https://doi.org/10.1037/emo0001097>

Lund, S. H., Bo, S., Bach, B., Jørgensen, M. S., & Simonsen, E. (2023). Mentalizing in Adolescents With and Without Prominent Borderline Features: Validation of the Reflective Functioning Questionnaire for Youths (RFQY) and an Investigation of the Factor Structure of Hypo- and Hypermentalizing. *Journal of Personality Assessment*, *105*(4), 475-486. <https://doi.org/10.1080/00223891.2022.2055474>

Matko, K., Sedlmeier, P., & Bringmann, H. C. (2022). Embodied cognition in meditation, yoga, and ethics - An experimental single-case study on the differential effects of four mind-body treatments. *International Journal of Environmental Research and Public Health*, *19*, 11734. <https://doi.org/10.3390/ijerph191811734>

McMahon, T. P., & Naragon-Gainey, K. (2019). The multilevel structure of daily emotion-regulation-strategy use: An examination of within- and between-person associations in naturalistic settings. *Clinical Psychological Science*, *7*, 321-339. <https://doi.org/10.1177/2167702618807408>

Medland, H., De France, K., Hollenstein, T., Mussoff, D., & Koval, P. (2020). Regulating Emotion Systems in Everyday Life: Reliability and Validity of the RESS-EMA Scale. *European Journal of Psychological Assessment*. <https://doi.org/10.1027/1015-5759/a000595>

Mestdagh, M., & Dejonckheere, E. (2021). Ambulatory assessment in psychopathology research: Current achievements and future ambitions. *Current Opinion in Pscychology*, *21*, 1-8. <https://doi.org/10.1016/j.copsyc.2021.01.004>

Nas, C. N., Brugman, D., & Koops, W. (2008). Measuring self-serving cognitive distortions with the "How I Think" Questionnaire. *European Journal of Psychological Assessment*, *24*(3), 181-189. <https://doi.org/10.1027/1015-5759.24.3.181>

Neumann, A., Van Lier, P. A. C., Gratz, K. L., & Koot, H. M. (2010). Multidimensional assessment of emotion regulation difficulties in adolescents using the Difficulties in Emotion Regulation Scale. *Assessment*, *17*, 138-149. <https://doi.org/10.1177/1073191109349579>

Norman-Nott, N., Wilks, C., Hesam-Shariati, N., Schroeder, J., Suh, J., Czerwinski, M., & Gustin, S. M. (2021). Efficacy of the iDBT-Pain skills training intervention to reduce emotional dysregulation and pain intensity in people with chronic pain: protocol for a single-case experimental design with multiple baselines. *BMJ Open*, *11*(4), e041745. <https://doi.org/10.1136/bmjopen-2020-041745>

Parkline Interactive LLC. (2019). *Wander [virtual reality application software]*. In (Version 0.3522) <https://www.meta.com/nl-nl/experiences/2078376005587859/?utm_source=www.parklineinteractive.com&utm_medium=oculusredirect>

Sauter, F. M., Heyne, D., Blote, A. W., van Widenfelt, B. M., & Westenberg, P. M. (2010). Assessing therapy-relevant cognitive capacities in young people: development and psychometric evaluation of the self-reflection and insight scale for youth. *Behav Cogn Psychother*, *38*(3), 303-317. <https://doi.org/10.1017/S1352465810000020>

Van der Helm, G. H., Wissink, I. B., De Jongh, T., & Stams, G. J. (2013). Measuring treatment motivation in secure juvenile facilities. *Int J Offender Ther Comp Criminol*, *57*(8), 996-1008. <https://doi.org/10.1177/0306624X12443798>

Van der Helm, G. H. P., De Jongh, T., & De Valk, S. (2017). Vragenlijst Behandelmotivatie voor Adolescenten. Handleiding. In *ATMQ: Adolescent Treatment Motivation Questionnaire*: Lectoraat Residentiële Jeugdzorg.

van Linschoten, R. C. A., Knijnenburg, S. L., West, R. L., & van Noord, D. (2023). CastorEDC API: A Python Package for Managing Real World Data in Castor Electronic Data Capture. *Journal of Open Research Software*, *11*(1), 12. <https://doi.org/https://doi.org/10.5334/jors.436>

Victor, S., & Klonsky, E. D. (2016). Validation of a brief version of the Difficulties in Emotion Regulation Scale in five samples. *Journal of Psychopathology and Behavioral Assessment*, *38*. <https://doi.org/10.1007/s10862-016-9547-9>
