# Supplementary material for: The First Step is the Hardest: A Mixed Methods Single-Case Experimental Design Study of a VR-Enhanced Training Program in a Forensic Youth Care Setting
Source: Res Child Adolesc Psychopathol. 2025 Apr 14;53(12):1733–53. doi: 10.1007/s10802-025-01313-1 (PMC12718268; doi:10.1007/s10802-025-01313-1)
Supplement: Supplementary file 2 — Supplementary Material 2 [file 10802_2025_1313_MOESM2_ESM.docx]

Appendix 2 – Results section

**Participants’ backgrounds and study trajectories**

*Jason*

Jason was a 16-year-old boy. In 2018 he was psychologically assessed and classified with Attention Deficit Hyperactivity Disorder (ADHD) and mild intellectual disability. ADHD medication in the form of methylphenidate was prescribed. Apart from starting at the ambulatory setting in 2022, he also started trauma therapy that year.

Jason was included in the study on January 10, 2023. Pretreatment assessment took place on January 17, which also marked the start of the baseline phase. On February 7, the first session of ST took place, meaning that the intervention phase started on this day. The second session took place on February 15. No sessions took place after this second one and Jason stopped completing measurements since. At this point, no contact was achieved with Jason. On March 9 the executive researcher was informed by his ST therapist that Jason had also stopped going to school, his internship, the ambulatory treatment setting, and his trauma therapy sessions; they also had lost contact with him.

On March 31, the researcher got back in touch with Jason, and he explained that he had been reported because he had violated his probation conditions. He spent a week on the run and was then arrested and detained for two weeks. He had reportedly just been released the day before. He stated being motivated to continue with the study and that he would start filling in the daily measurements again. He did complete the measurement of that day, however, the following two weeks no more measurements were completed. On April 12, the researcher got in contact with his ST therapist. It appeared Jason had not been back to the treatment setting and that his story about what had happened in between was untrue. Attempts to resume execution were unsuccessful. Finally, on June 27, the researcher was able to officially close off the study together with Jason.

*ST sessions*

During the first session, the goal of ST was explained to Jason and the program was discussed. As this was the first ST session that was done for this study setting, the researcher was present to resolve possible questions or issues. The first exercise was done, leading to the formulation of a personal learning goal. It was not managed to introduce VR, as the VR headset was stored in a locked cabinet that the ST therapist did not have the key to at that moment.

During the second session, the VR video was viewed, and Jason choose one of the characters for the exercises. Jason and his ST therapist collaboratively created a backstory for the character and continued with viewing the VR video again, paying specific attention to this character in the video. The ST therapist got the idea that in creating the backstory, Jason used a lot of fantasy at first and then started talking more and more about himself. After the backstory was finished, they continued with the perspective-taking exercises. The ST therapist reported the session was successful, although the startup was difficult. She was not entirely accustomed to ST and VR yet and it took several attempts to stream the footage from the VR headset to the laptop so she could watch along. She called the researcher about this during the session and via phone it was managed to resolve the problem. After thirty minutes Jason’s concentration decreased, but he was positive about the session.

*Steve*

Steve was another 16-year-old boy. In 2019 he followed Training Aggression Controle treatment (TACt) and received a supervision and guidance measure from a juvenile probation officer. In 2020 a forensic psychological assessment was done and he was classified with ADHD and mild intellectual disability. In 2022 he was diagnosed with post-traumatic stress disorder (PTSD). Trauma therapy was started (Eye Movement and Desensitization and Reprocessing; EMDR), as well as multisystemic therapy (MST) because of family problems. Moreover, he started with Aggression Regulation Therapy.

Steve was included in the study on February 13, 2023, and pretreatment assessment took place on the same day, also marking the start of the baseline phase. The baseline phase ended on February 21, as the first ST sessions took place on February 22. Not all sessions lasted between 45 and 60 minutes and it proved unfeasible to conduct two sessions per week. This was due to practical constraints, such as Steve being late for his appointment, because he also attended school, or because of his community service. Steve’s study period also included a holiday.

On March 21 it became clear Steve’s phone had broken, explaining the two-week gap in terms of completed measurements during the first half of the intervention phase. Steve himself could not quickly arrange for a new phone to continue the measurements, so we arranged a temporary phone for him with which he could continue completing the daily measurements. Steve received the phone on March 24, and we restored the m-Path app using the recovery code. That day he resumed completing the measurements. The last ST session took place on April 17. The last follow-up measurement was conducted on April 26, and the researcher and Steve could close off the study period together on May 11. Steve declined to participate in the long-term follow-up interviews 3 and 6 months later.

*ST sessions*

During the first session, Steve enjoyed playing with the VR headset as an introduction to VR. Based on the first exercise, his learning goal was to listen more to another person’s thoughts and feelings for example, in order to understand this person better. In the following sessions of module 1 there were struggles to connect the VR headset to the laptop, which obstructed streaming multiple times. Nonetheless, Steve enjoyed creating backstories for the different characters and reflecting on the scenario from different perspectives. During the last session of module 1, however, it seemed like the same exercises with the same scenario started to get boring for Steve.

In module 2, the streaming problems kept coming back. Therefore, instead of watching along with what Steve was seeing inside the headset, his ST therapist watched along on Google street view on a smartphone when Steve visualized his personal experience. Steve was impressed to virtually return to the location of his experience, having not returned to that location since the incident had happened a few years earlier. Character building went very well and the perspective switching exercises were also very entertaining because the ST therapist was familiar with one of the chosen personal characters. Moreover, by switching perspectives with one of the bystanders during the incident, Steve realized that he had never apologized and that perhaps he should still do so because it probably must have been very intense for this person. During the last session, Steve and his ST therapist discussed that a different learning goal would fit better now; instead of the other way around, he now wished to learn to explain to others what he thinks, feels, or would like to do in a certain situation, so others could better understand him.

*Aron*

Aron was a 17-year-old boy. In November 2023 he underwent a psychological assessment because of a suspected offence, by order of the district attorney. At the ambulatory setting he did a ‘social environment analysis’, related to the same offence, together with the same therapist he did ST with.

Aron was included in the study on October 12, 2023, and pretreatment assessment took place on October 13, which marked the start of the baseline phase. The first ST session took place on October 30. For Aron it was sometimes managed to conduct two ST sessions per week, but again it proved unfeasible not to skip weeks in between. The study period again included a holiday, and appointments were sometimes canceled due to practical reasons.

During the baseline phase, Aron was relatively faithful in completing the daily measurements, which changed when the intervention phase started. At first, in November, it was still managed to get in touch with Aron about this and he indicated that he kept forgetting to fill in the questionnaires. However, in December Aron was no longer reachable and it appeared he had a new phone number. It was managed to get in touch with him again, but a few days after the last contact it became clear Aron had been arrested. He was detained for a few weeks, which also meant that he did not have a phone at his disposal to fill in the questionnaires. At this time, the first module of ST was finished. On January 23, 2024, the execution of ST was resumed. Nevertheless, Aron did not resume to complete daily measurements until post-measurement. The last ST session took place on February 5. The last follow-up measurement was conducted on February 13, and the researcher and Aron could close off the study period together on the same day. The long-term follow-up interviews at 3- and 6 months follow-up have not been conducted, because data-collection had ended beforehand.

*ST sessions*

Aron’s ST therapist did not complete the ST sessions forms, despite continuous reminders. Therefore, it is unclear on what days the sessions exactly have been conducted. The information described here, is based on the contact moments with Aron and his ST therapist during the study period.

After the first session, Aron’s ST therapist mentioned she was under the impression that Aron liked it so far. During the second session, the first character from the video was addressed. Following, it took a while to conduct the third session because of an incident that required attention first, according to the involved youth protection worker. Mid-December the third and fourth session were done, during which technical problems with the VR video arose; the video kept stalling when playing. It was not managed to fix this problem during the session, because of which they were not able to fully complete the exercises since Aron had a curfew he had to abide by.

After the fourth session Aron got arrested. When continuing in January with the last couple of sessions, Aron’s ST therapist mentioned that Aron really enjoyed the second ST module in which he could talk about his own experiences and show her where these took place. She herself also enjoyed watching along with his visualizations. Streaming, however, did not go flawless. During one session the Wander app did not work properly at first, leading to delay.
