# Supplementary material for: The First Step is the Hardest: A Mixed Methods Single-Case Experimental Design Study of a VR-Enhanced Training Program in a Forensic Youth Care Setting
Source: Res Child Adolesc Psychopathol. 2025 Apr 14;53(12):1733–53. doi: 10.1007/s10802-025-01313-1 (PMC12718268; doi:10.1007/s10802-025-01313-1)
Supplement: Supplementary file 3 — Supplementary Material 3 [file 10802_2025_1313_MOESM3_ESM.docx]

Appendix 3 – Results Jason

**Daily repeated measurements Jason**

**Figure 1**

*Item 1. How are you feeling right now? – ☹ (0) 🡪 ☺ (100)*


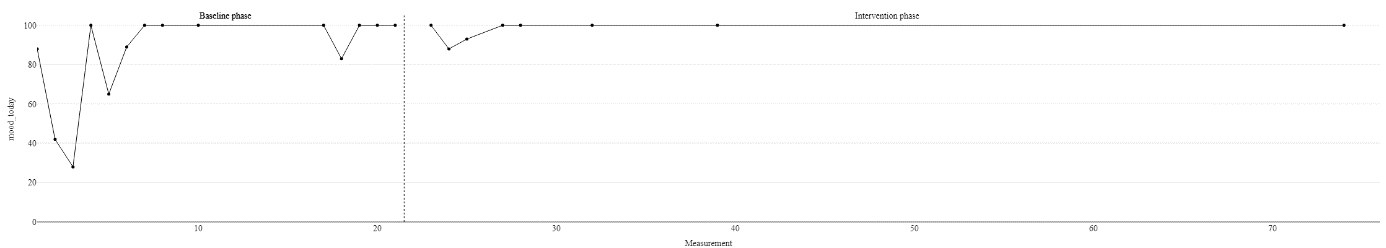
When looking at item 1, it was visible that the baseline scores were more scattered than those in the intervention phase. The baseline phase contained several lower scores, which did not recur in the intervention phase. Intervention scores remained high, also after the last ST session (day 30). However, scores already improved in the first half of the baseline phase, meaning that it could not be established that the intervention was likely to have caused the improvement. It may be that improvement was partly due to participation in itself, or to daily reflection by completing the questionnaires, for example.

**Figure 2**

*
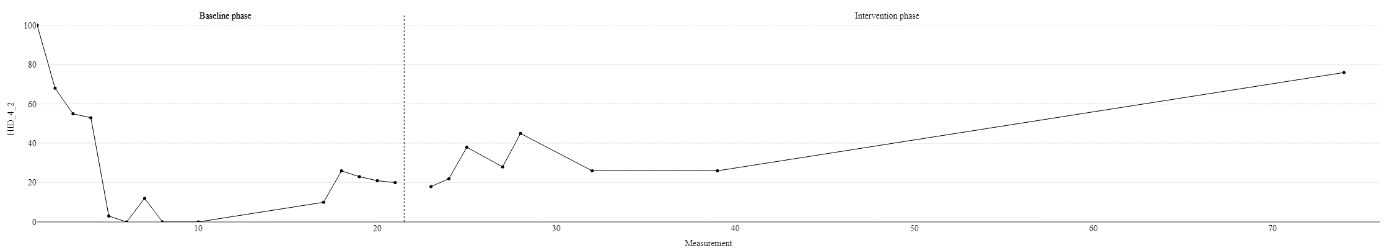
Item 2. If I lose my temper, there is nothing I can do about it – totally disagree (0) 🡪 totally agree (100)*

**Figure 3**

*Item 3. No matter how hard I try, I can’t help getting in trouble today – totally disagree (0) 🡪 totally agree (100)*


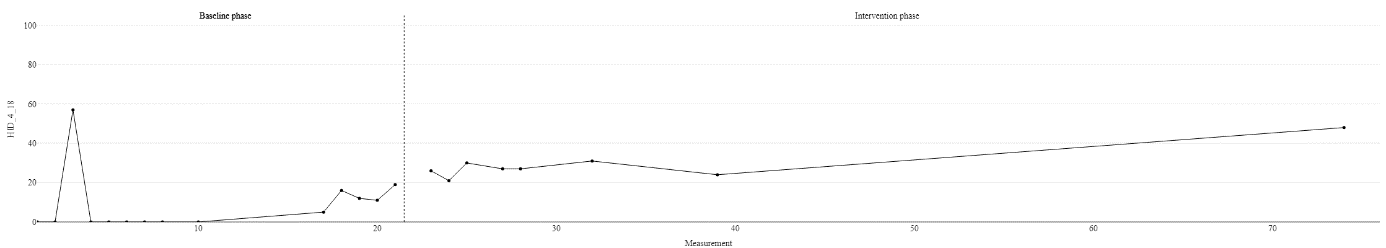


**Figure 4**

*
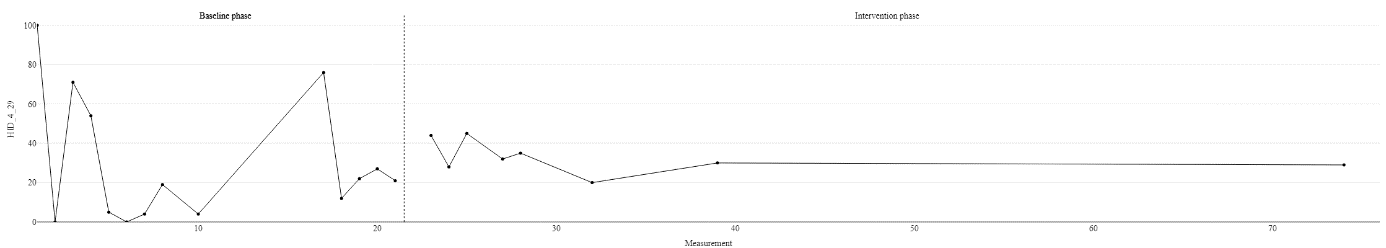
Item 4. People tried to bother me today – totally disagree (0) 🡪 totally agree (100)*

Regarding cognitive distortions, represented by items 2, 3, and 4, it was expected that scores would decrease throughout the study period. For all items, a similar pattern to item 1 was observed. The baseline phase of all three items showed more variation in scores than those in the intervention phase, which followed a more stable course. Items 2 and 3 showed a rather unfavorable trend, whereby the low (more positive) scores from the baseline phase did not recur in the intervention phase. Furthermore, concerning these two items, it was visible that, just before dropout, the scores increased again. Item 4, assessing whether Jason perceived other people to try and purposefully bother him that day, showed a more favorable trend. Although the lowest scores from the baseline did not recur during the intervention, the high peaks did not recur either. Therefore, comparing baseline to intervention, it seemed that Jason both did and did not interpret other people as trying to get in his way during baseline, whereas he less interpreted other people as such during the intervention phase.

**Figure 5**

*
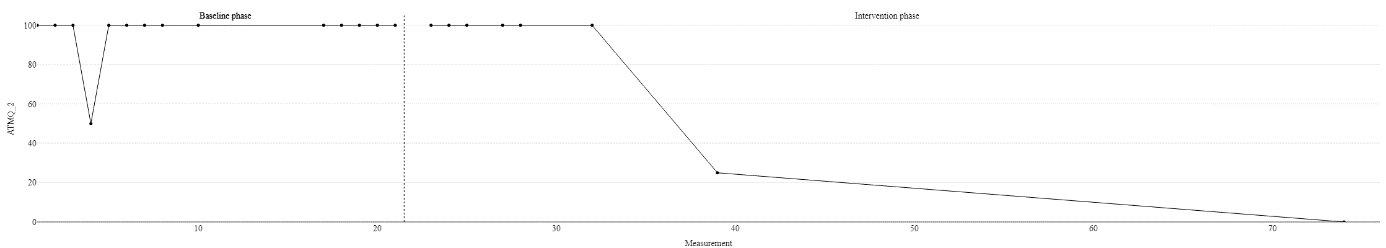
Item 5. I find that my guidance here is useful – not true (0) 🡪 true (100)*

**Figure 6**

*Item 6. I talked about myself with my counselors today – not true (0) 🡪 true (100)*


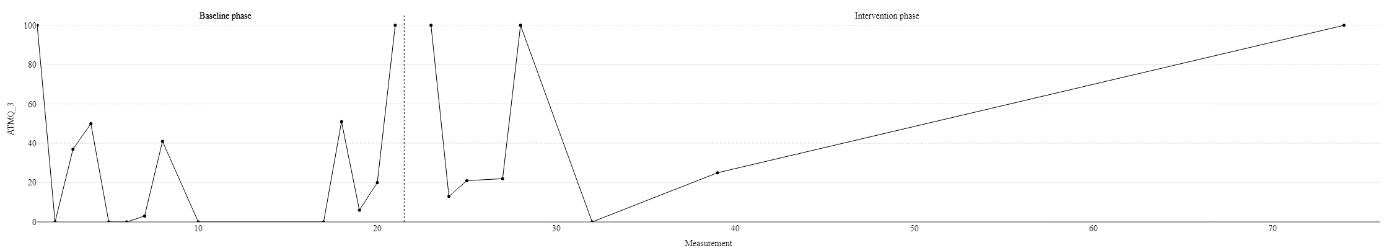


**Figure 7**

*
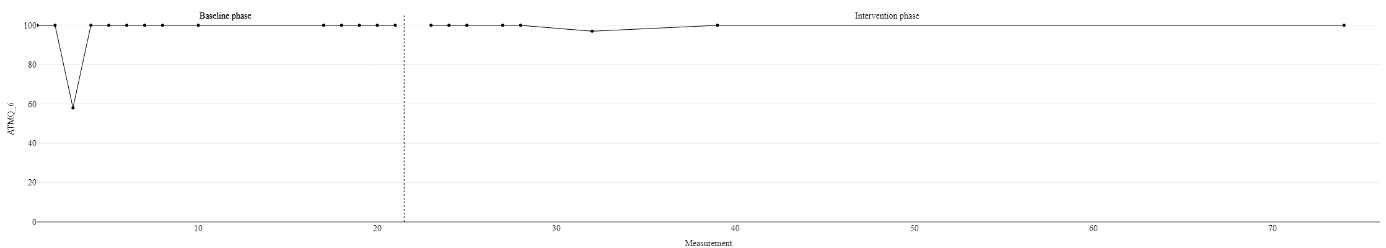
Item 7. I trust my counselors – not true (0) 🡪 true (100)*

**Figure 8**

*
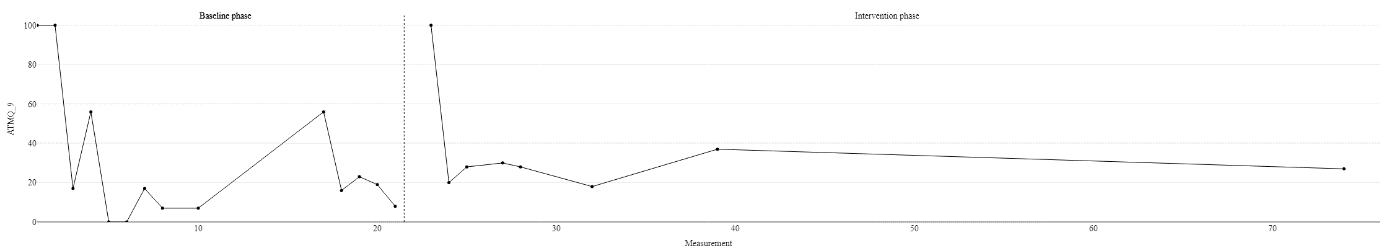
Item 8. I thought about my behavior today – not true (0) 🡪 true (100)*

**Figure 9**

***
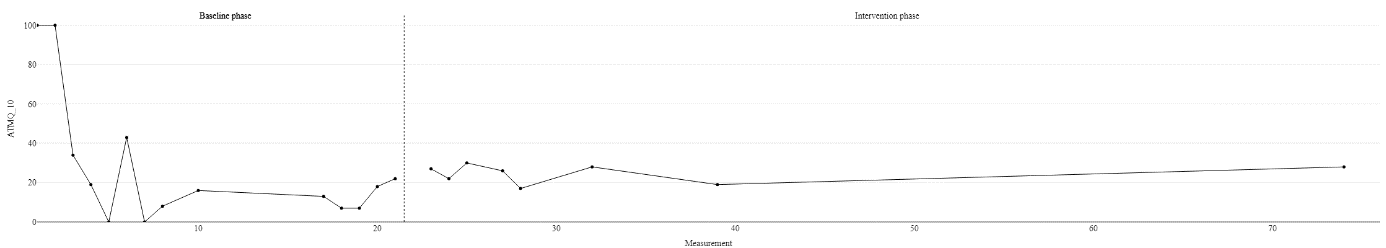
****Item 9. I want to change my behavior together with others – not true (0) 🡪 true (100)*

Looking at motivation, represented by items 5 through 9, varying results were visible. Scores were expected to be higher during intervention, compared to baseline. The patterns of items 5, 8, and 9 fitted Jason’s dropout and did not show a positive effect of the intervention. Item 6, assessing whether Jason talked about himself with his counselors that day, was rather difficult to interpret. The variation is explicable, since Jason did not see his counselors every day and thus did not talk to them every day. Item 7 showed a favorable pattern in general, considering that Jason reported to trust his counselors from the start on. The lowest score during baseline did not recur during intervention, despite other deteriorations and dropping out.

**Figure 10**

*Item 10. In response to my emotions today, I looked at things from a different angle – not true (0) 🡪 true (100)*


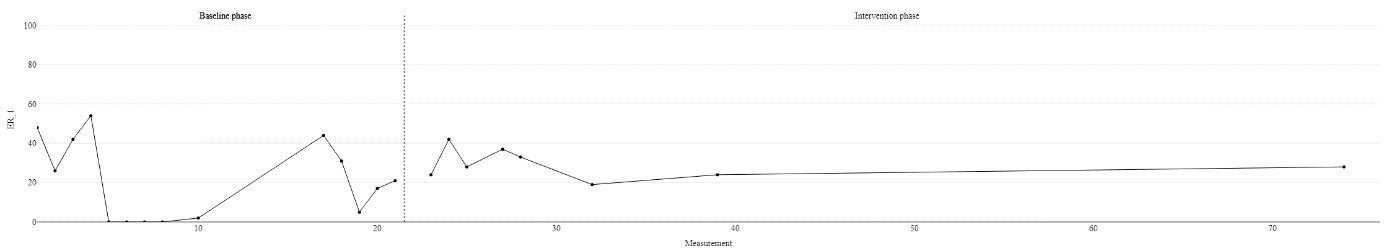
**Figure 11**

*
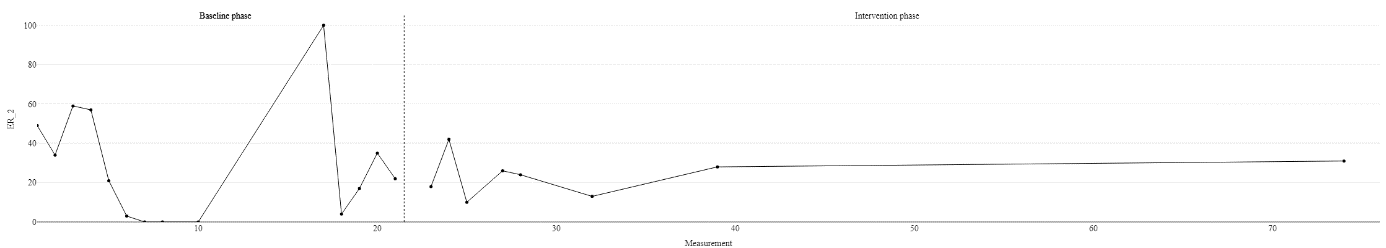
Item 11. When I am upset, I lose control over my behavior – not true (0) 🡪 true (100)*

Regarding emotion regulation, illustrated by items 10 and 11, we expected scores to increase for item 10 and to decrease for item 11. We could be cautiously positive about the difference between baseline and intervention. Both items showed a positive trend, with a more stable pattern during the intervention phase. For item 10, the lowest scores did not return in the intervention phase, but neither did the highest scores. For item 11, it appeared Jason perceived to have more control over his behavior when he would get upset throughout the intervention phase, also towards dropping out.
