# Supplementary material for: The First Step is the Hardest: A Mixed Methods Single-Case Experimental Design Study of a VR-Enhanced Training Program in a Forensic Youth Care Setting
Source: Res Child Adolesc Psychopathol. 2025 Apr 14;53(12):1733–53. doi: 10.1007/s10802-025-01313-1 (PMC12718268; doi:10.1007/s10802-025-01313-1)
Supplement: Supplementary file 4 — Supplementary Material 4 [file 10802_2025_1313_MOESM4_ESM.docx]

Appendix 4 – Results Steve

**Daily repeated measurements Steve**

**Figure 1**

*
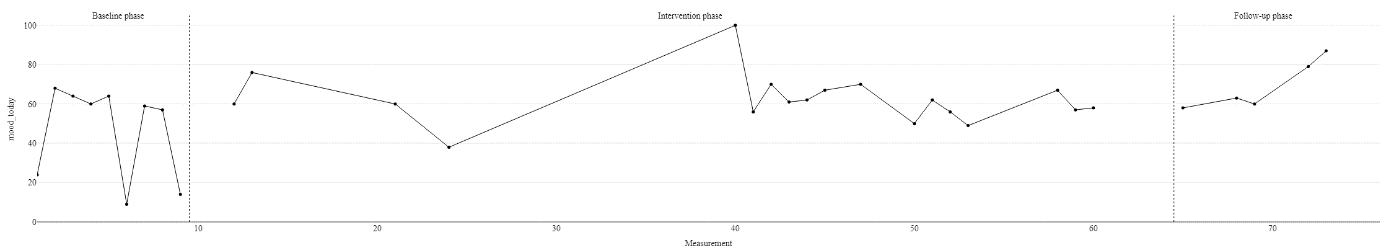
Item 1. How are you feeling right now? – ☹ (0) 🡪 ☺ (100)*

Regarding Steve’s mood throughout the study period, a cautiously positive trend was visible from baseline through follow-up, indicating that Steve started to feel better. The lowest scores from the baseline phase did not recur in the intervention phase, and the lowest scores from the intervention phase did not return in the follow-up phase.

**Figure 2**

*
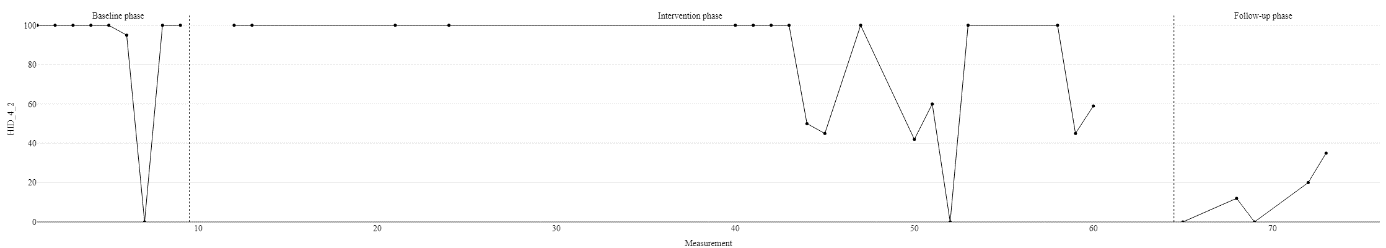
Item 2. If I lose my temper, there is nothing I can do about it – totally disagree (0) 🡪 totally agree (100)*

**Figure 3**

*Item 3. No matter how hard I try, I can’t help getting in trouble today – totally disagree (0) 🡪 totally agree (100)*


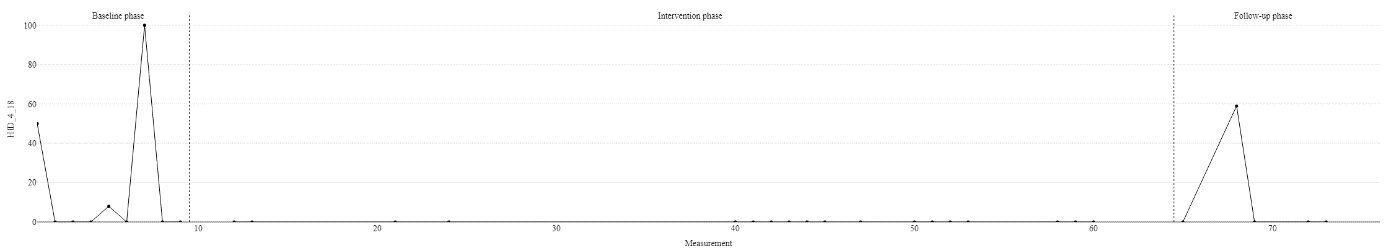


**Figure 4**

*Item 4. People tried to bother me today – totally disagree (0) 🡪 totally agree (100)*


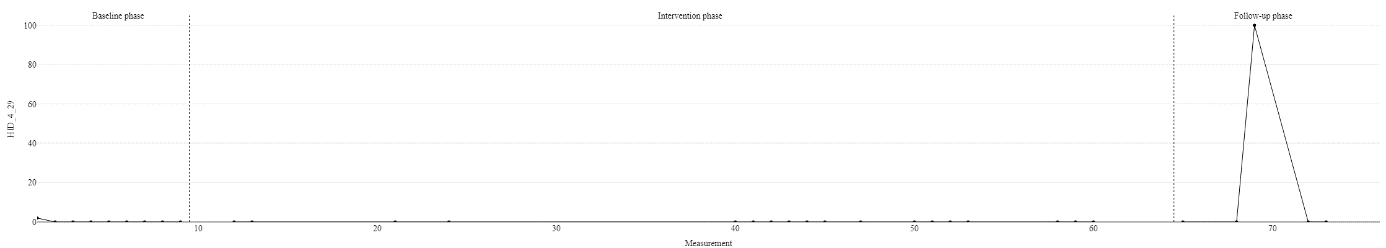
For cognitive distortions, it was expected that scores would decrease. Especially item 2 showed an interesting pattern. Apart from one outlier in the baseline phase, the scores were all as high as possible throughout the first half of the intervention phase, indicating that Steve totally agreed with the statement. Then, the scores started to fluctuate, exactly when Steve transitioned from module 1 to 2; the last session of module 1 was on day 38, the first of module 2 on 45. Steve started to agree less with the statement that there is nothing he can do about losing his temper, and this trend continued in the follow-up phase. In this last phase no high scores were reported anymore. Regarding item 3, the intervention phase only showed totally disagreeing scores. This was a positive contrast with the baseline and follow-up phases, which did show agreeing scores. For item 4 a ceiling effect was visible, as the scores were already as positive as possible from the start of the baseline. This continued through follow-up, apart from one outlying value.

**Figure 5**

*
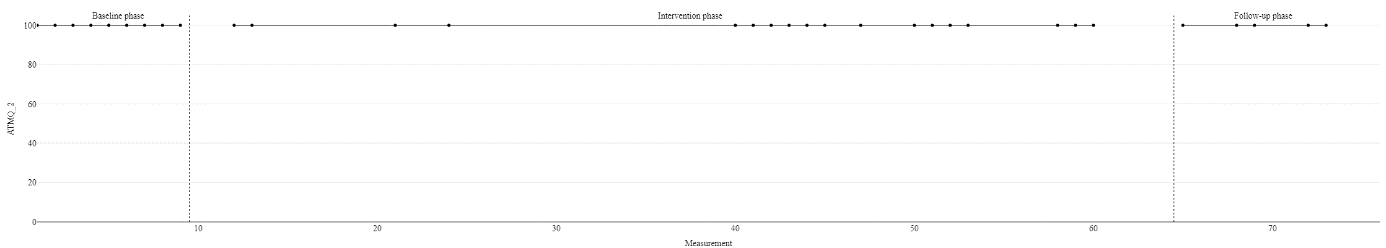
Item 5. I find that my guidance here is useful – not true (0) 🡪 true (100)*

**Figure 6**

*
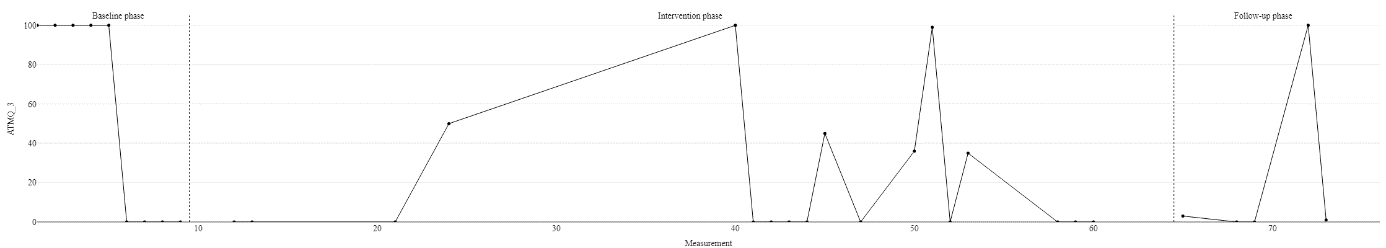
Item 6. I talked about myself with my counselors today – not true (0) 🡪 true (100)*

**Figure 7**

*Item 7. I trust my counselors – not true (0) 🡪 true (100)*


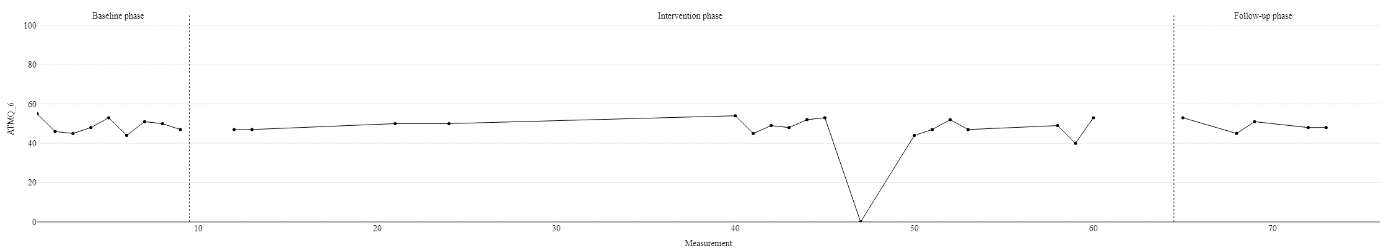


**Figure 8**

*
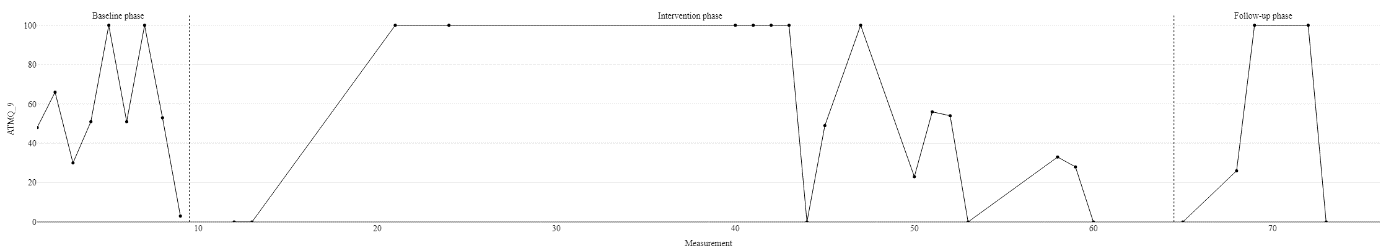
Item 8. I thought about my behavior today – not true (0) 🡪 true (100)*

**Figure 9**

*Item 9. I want to change my behavior together with others – not true (0) 🡪 true (100)*


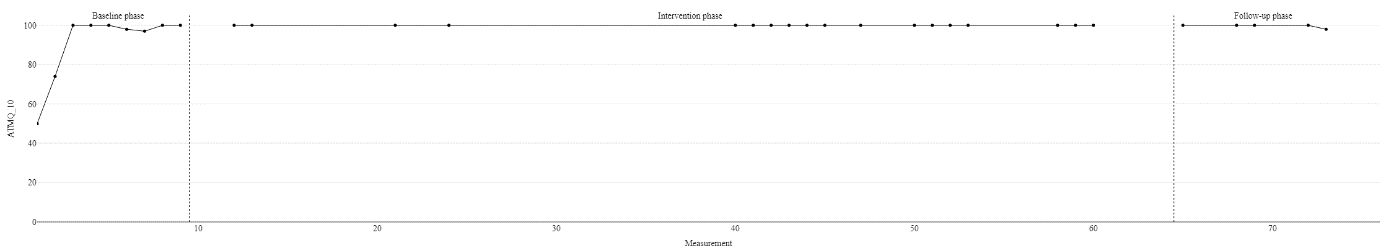
For motivation, it was expected that scores would be higher during the intervention phase, compared to the baseline phase. From the start on, Steve reported he finds his guidance at the treatment setting useful (item 5). As with item 4 a ceiling effect was visible, and the scores did not change. The same could be concluded for item 9, although the first two scores were lower. Regarding item 7, no visible change could be detected when looking at the pattern. The outlier in the intervention phase for this item was notable. For items 6 and 8, a fairly similar pattern as that of item 2 could be distilled, with fluctuations in the second half of the intervention phase. Regarding item 8, assessing whether Steve thought about his behavior that day, it may have been the case that this was too confrontational in relation to discussing his personal experience in module 2, in which he acted as a perpetrator. However, the question remains to what extent this was consciously considered.

**Figure 10**

*Item 10. In response to my emotions today, I looked at things from a different angle – not true (0) 🡪 true (100)*


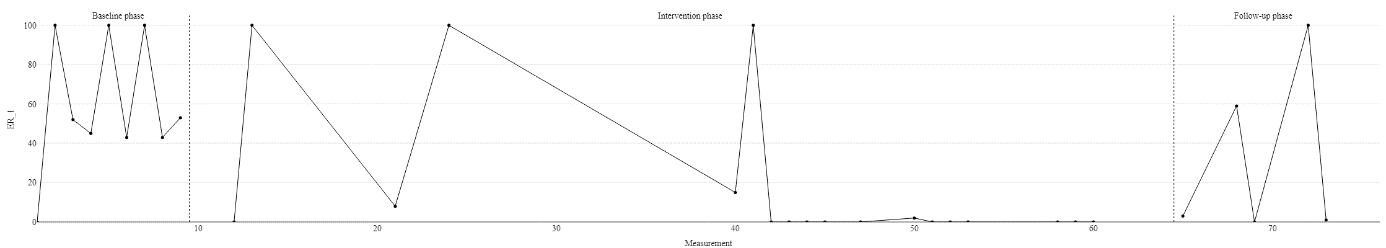
**Figure 11**

*
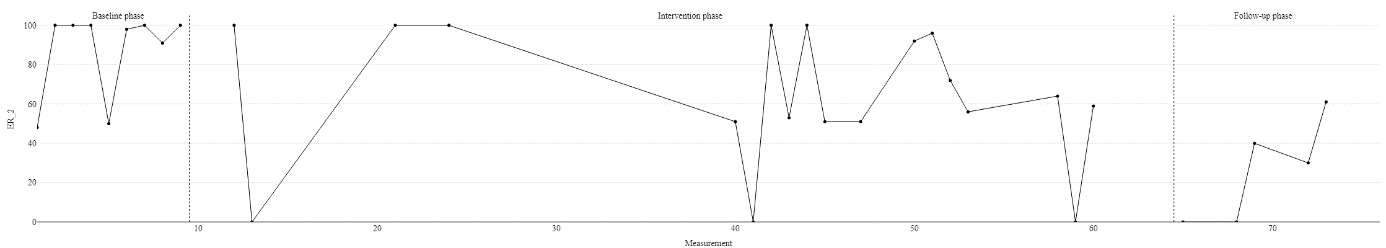
Item 11. When I am upset, I lose control over my behavior – not true (0) 🡪 true (100)*

The outcomes for the two emotion regulation items showed considerable spread and fluctuation. We expected scores to increase for item 10 and to decrease for item 11. For both items a similar pattern as that of items 2, 6 and 8 could be observed, with deviating or fluctuating scores in the second half of the intervention phase. Again, the confrontation of discussing his personal experience may have played a role in this. Regarding item 11 specifically, Steve appeared to perceive more control over his behavior when he would get upset at this point. This seemed to be carried over into the follow-up phase.

**Pre-, post-, and follow-up assessment Steve**

**Table 3**

*How I Think questionnaire*

|  | AR^a^ | SC^b^ | BO^c^ | MM^d^ | AW^e^ | OD^f^ | PA^g^ | L^h^ | S^i^ | OV^j^ | CV^k^ | HIT^l^ |
| --- | --- | --- | --- | --- | --- | --- | --- | --- | --- | --- | --- | --- |
| Pre | 3,38 | 2,22 | 2,2 | 2,22 | 2,55 | 2,5 | 2,6 | 3,13 | 1,27 | 2,55 | 2,2 | 2,34 |
| Post | 3,5 | 2,56 | 3,2 | 2,56 | 2,55 | 2,7 | 3 | 2,88 | 2,36 | 2,85 | 2,62 | 2,73 |
| Follow-up | 2,87 | 2,33 | 2,9 | 2,33 | 1,91 | 2,7 | 2,6 | 2,25 | 1,91 | 2,65 | 2,08 | 2,37 |

*Note. ^a^*AR = Anomalous response; ^b^SC = Self-centered; ^c^BO = Blaming others; ^d^MM = Minimizing mislabeling; ^e^AW = Assuming the worst; ^f^OD = Oppositional defiant; ^g^PA = Physical aggression; ^h^L = Lying; ^i^S = Stealing; ^j^OV = Overt behavior; ^k^CV = Covert behavior; ^l^HIT = How I Think total score; green = non-clinical range; orange = borderline clinical range; red = clinical range.

**Table 4**

*ATMQ*

| ATMQ |  |  |  |
| --- | --- | --- | --- |
|  | Score |  | RCI |
| Pre | 2,09 | Pre – post | 0,82 |
| Post | 2,36 | Post – follow-up | -1,37 |
| Follow-up | 1,91 | Pre – Follow-up | -0,55 |

*Note.* ATMQ = Adolescent Treatment Motivation Questionnaire.

**Table 5**

*RFQY*

| RFQY |  |  |  |
| --- | --- | --- | --- |
|  | Score |  | RCI |
| Pre | 7,04 | Pre – post | 1,70 |
| Post | 8,23 | Post – follow-up | -0,83 |
| Follow-up | 7,65 | Pre – Follow-up | 0,87 |

*Note.* RFQY = Reflective Functioning Questionnaire for Youths.

**Table 6**

*SRIS-Y*

| SRIS-Y |  |  |  |  |  |  |  |
| --- | --- | --- | --- | --- | --- | --- | --- |
|  | Score SR |  | RCI SR |  | Score I |  | RCI I |
| Pre | 29 | Pre – post | 3,85* | Pre | 11 | Pre – post | 3,21* |
| Post | 32 | Post – follow-up | 2,57* | Post | 14 | Post – follow-up | -4,28* |
| Follow-up | 34 | Pre – Follow-up | 6,42* | Follow-up | 10 | Pre – Follow-up | -1,07 |

*Note.* SRIS-Y = Self-Reflection and Insight Scale for Youth; * = RCI exceeds (-)1.96.

**Table 7**

*PT-IRI*

| PT-IRI |  |  |  |
| --- | --- | --- | --- |
|  | Score |  | RCI |
| Pre | 4 | Pre – post | 2,53* |
| Post | 12 | Post – follow-up | 0,63 |
| Follow-up | 14 | Pre – Follow-up | 3,16* |

*Note.* PT-IRI = Perspective Taking subscale of the Interpersonal Reactivity Index; * = RCI exceeds (-)1.96.
