# Supplementary material for: The First Step is the Hardest: A Mixed Methods Single-Case Experimental Design Study of a VR-Enhanced Training Program in a Forensic Youth Care Setting
Source: Res Child Adolesc Psychopathol. 2025 Apr 14;53(12):1733–53. doi: 10.1007/s10802-025-01313-1 (PMC12718268; doi:10.1007/s10802-025-01313-1)
Supplement: Supplementary file 5 — Supplementary Material 5 [file 10802_2025_1313_MOESM5_ESM.docx]

Appendix 5 – Results Aron

**Daily repeated measurements Aron**

**Figure 1**

***
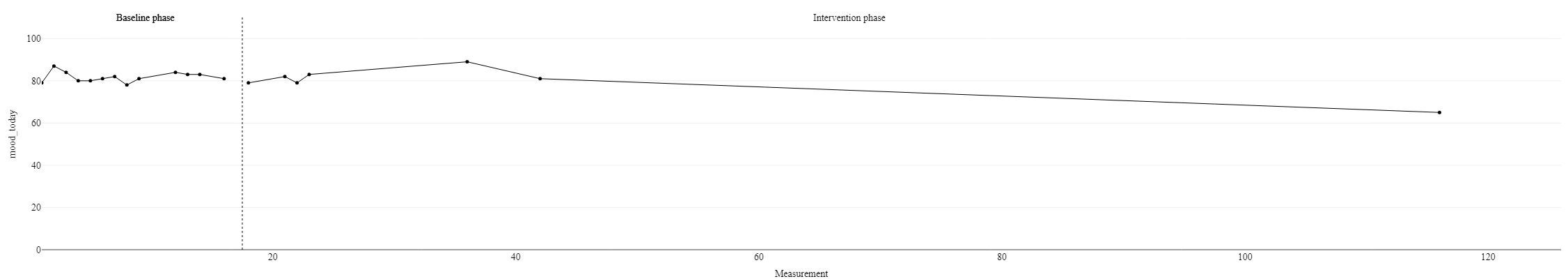
****Item 1. How are you feeling right now? – ☹ (0) 🡪 ☺ (100)*

Aron’s mood did not appear to have changed throughout the study period, looking at the baseline phase and the first part of the intervention phase. He quite steadily reported to feel good, which also lead to a ceiling effect. However, the last completed measurement indicated a slight decrease. This may be related to the period in between, during which he was detained.

**Figure 2**

*Item 2. If I lose my temper, there is nothing I can do about it – totally disagree (0) 🡪 totally agree (100)*


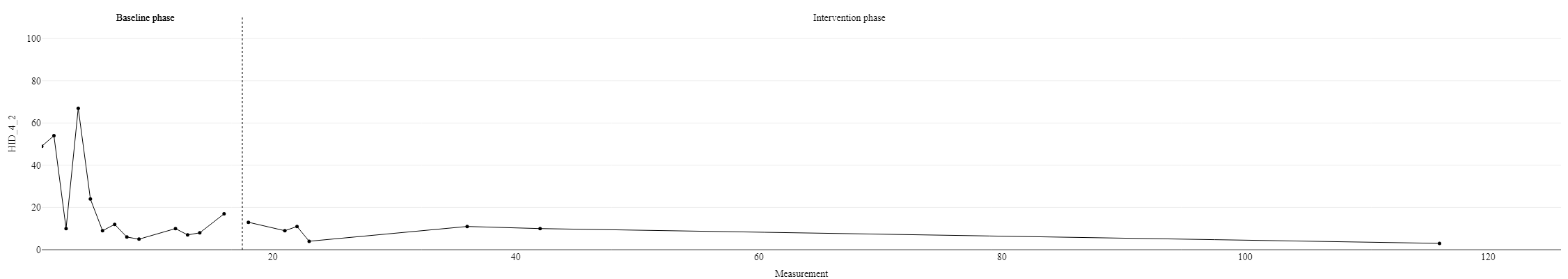
**Figure 3**

*
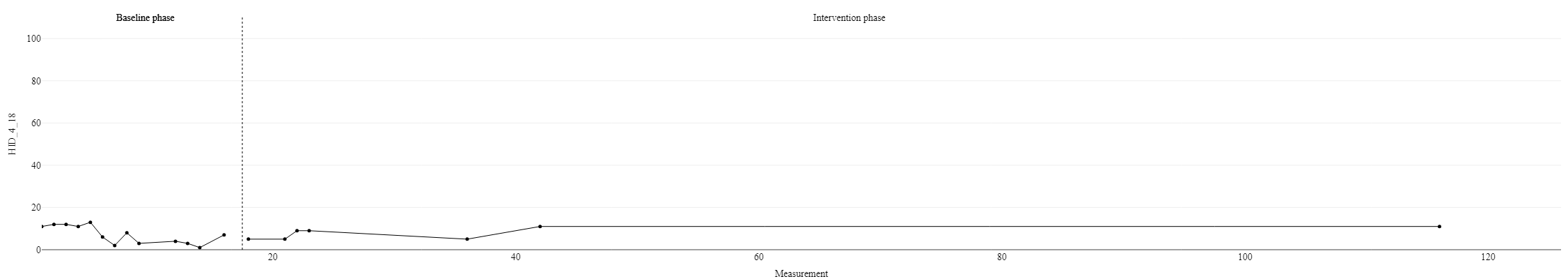
Item 3. No matter how hard I try, I can’t help getting in trouble today – totally disagree (0) 🡪 totally agree (100)*

**Figure 4**

*Item 4. People tried to bother me today – totally disagree (0) 🡪 totally agree (100)*


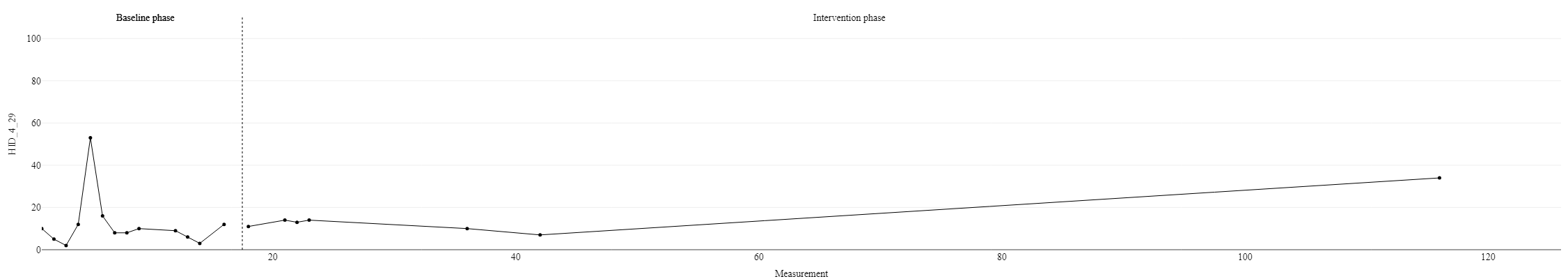


Regarding cognitive distortions, we expected a decline in scores throughout the study period. Different patterns could be established. Item 3 showed a similar pattern as that of item 1. The baseline phase already showed little room for improvement, and scores remained low throughout the intervention phase. Items 2 and 4 showed some variability in the beginning, but already during the baseline phase scores declined. They stayed low during the intervention phase, apart from item 4’s last measurement.

**Figure 5**

*
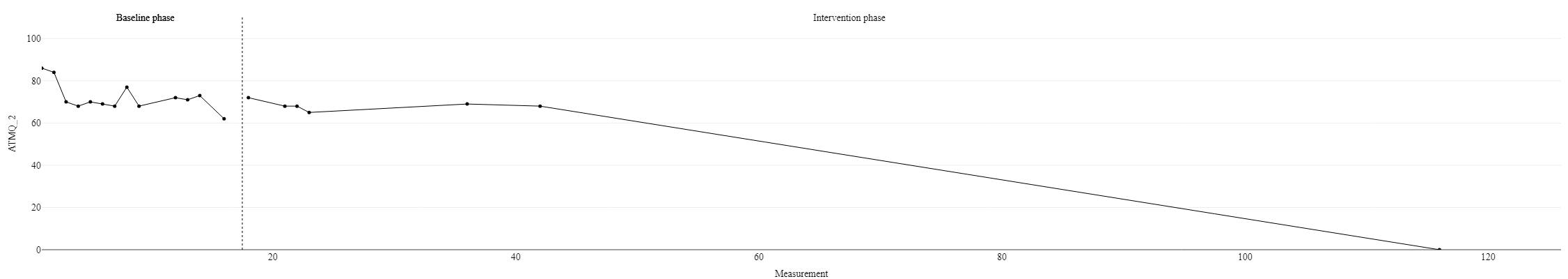
Item 5. I find that my guidance here is useful – not true (0) 🡪 true (100)*

**Figure 6**

*
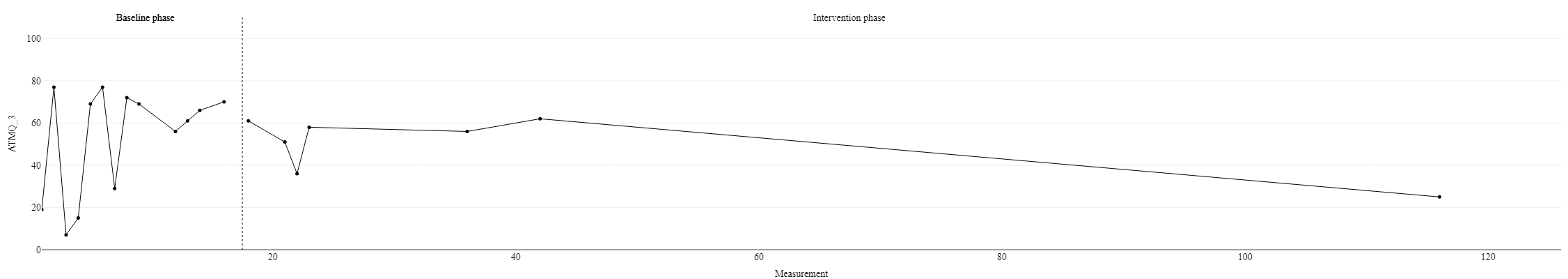
Item 6. I talked about myself with my counselors today – not true (0) 🡪 true (100)*

**Figure 7**

*Item 7. I trust my counselors – not true (0) 🡪 true (100)*


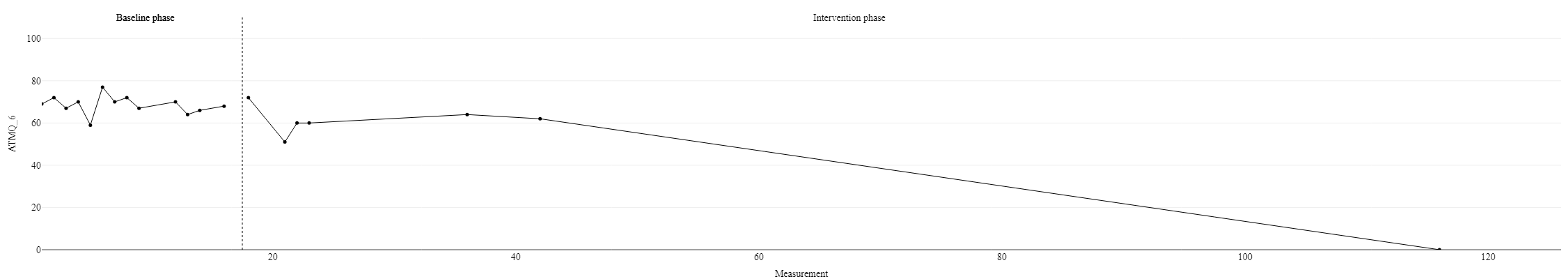
**Figure 8**

*
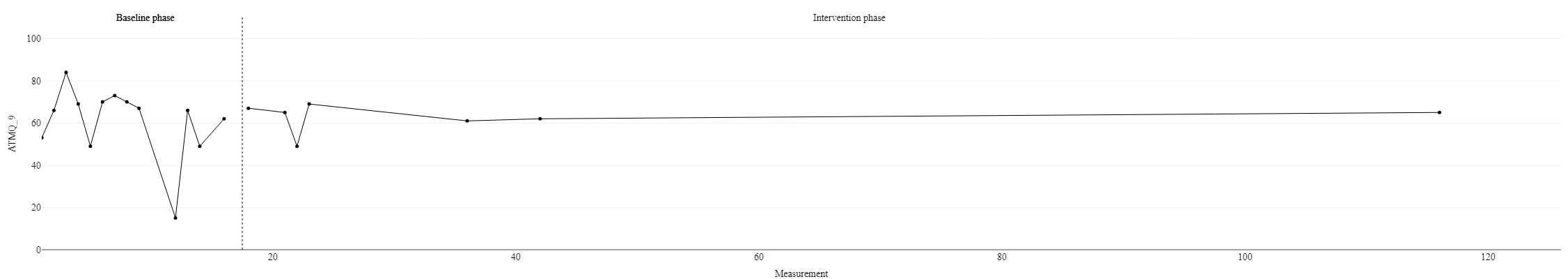
Item 8. I thought about my behavior today – not true (0) 🡪 true (100)*

**Figure 9**

*
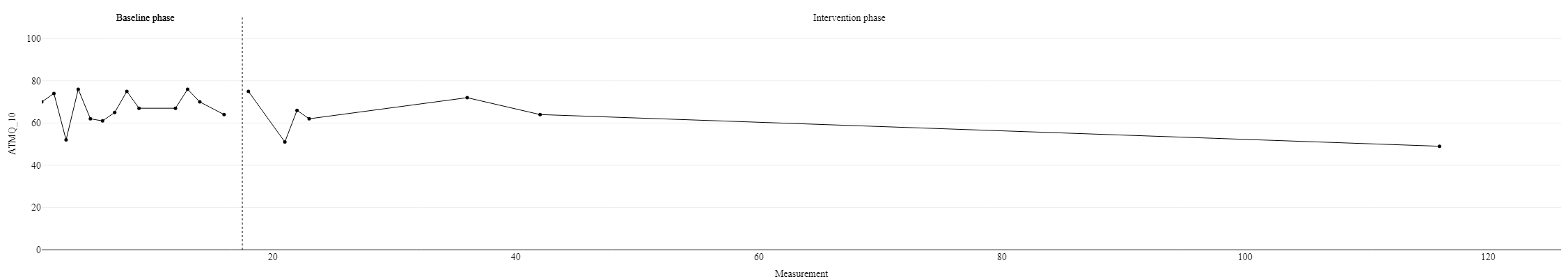
Item 9. I want to change my behavior together with others – not true (0) 🡪 true (100)*

The scores for motivation (items 5 through 9) were expected to increase throughout the study period. However, for all but one item (number 8) a decline in scores was visible at the end of the intervention phase. The scores of items 5 and 7 dropped completely, items 6 and 9 showed less decline. Apart from this drop, it was also visible that the baseline phases of the items showed more variability than the intervention phases. Most items showed more stability during the intervention phase, also meaning that higher scores from the baseline phases did not, or hardly, recur in the intervention phases.

**Figure 10**

*
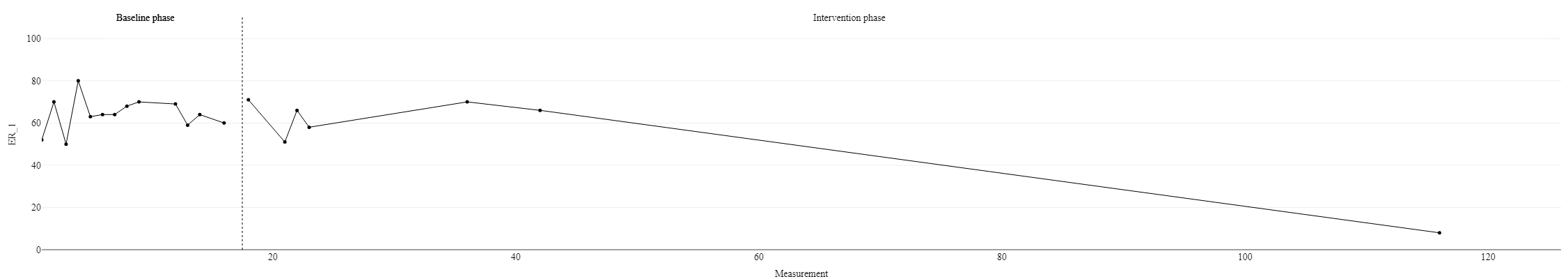
Item 10. In response to my emotions today, I looked at things from a different angle – not true (0) 🡪 true (100)*

**Figure 11**

*Item 11. When I am upset, I lose control over my behavior – not true (0) 🡪 true (100)*


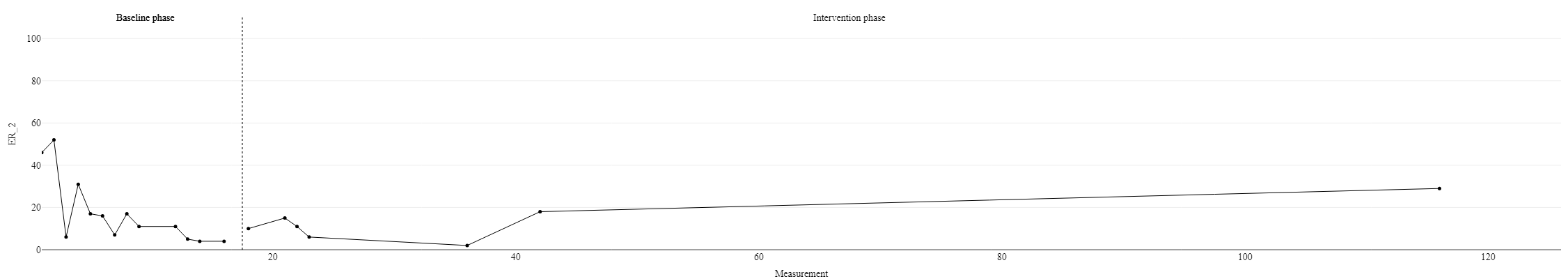


Regarding emotion regulation, item 10 was expected to show an increase in scores and item 11 to show a decrease. Little to no change for item 10 was visible, apart from the last score that was much lower compared to the rest. Item 11 showed a slightly more positive pattern, although it remains unclear to what extent this can be attributed to the intervention, since the change was already observable during the baseline phase.

**Pre-, post-, and follow-up assessment Aron**

**Table 8**

*How I Think questionnaire*

|  | AR^a^ | SC^b^ | BO^c^ | MM^d^ | AW^e^ | OD^f^ | PA^g^ | L^h^ | S^i^ | OV^j^ | CV^k^ | HIT^l^ |
| --- | --- | --- | --- | --- | --- | --- | --- | --- | --- | --- | --- | --- |
| Pre | 2,25 | 2,33 | 2,7 | 2 | 2,72 | 2,8 | 2,5 | 2,38 | 2,18 | 2,65 | 2,28 | 2,45 |
| Post | 2,75 | 2,33 | 2 | 2 | 2 | 2 | 2 | 2,34 | 2 | 2 | 2,17 | 2,08 |
| Follow-up | 3,5 | 2 | 2 | 2 | 2 | 2 | 2 | 2 | 2 | 2 | 2 | 2 |

*Note. ^a^*AR = Anomalous response; ^b^SC = Self-centered; ^c^BO = Blaming others; ^d^MM = Minimizing mislabeling; ^e^AW = Assuming the worst; ^f^OD = Oppositional defiant; ^g^PA = Physical aggression; ^h^L = Lying; ^i^S = Stealing; ^j^OV = Overt behavior; ^k^CV = Covert behavior; ^l^HIT = How I Think total score; green = non-clinical range; orange = borderline clinical range; red = clinical range.

**Table 9**

*ATMQ*

| ATMQ |  |  |  |
| --- | --- | --- | --- |
|  | Score |  | RCI |
| Pre | 2,27 | Pre – post | 0,55 |
| Post | 2,45 | Post – follow-up | 1,68 |
| Follow-up | 3 | Pre – Follow-up | 2,22* |

*Note.* ATMQ = Adolescent Treatment Motivation Questionnaire ; * = RCI exceeds (-)1.96.

**Table 10**

*RFQY*

| RFQY |  |  |  |
| --- | --- | --- | --- |
|  | Score |  | RCI |
| Pre | 7,74 | Pre – post | -0,06 |
| Post | 7,7 | Post – follow-up | 0,93 |
| Follow-up | 8,35 | Pre – Follow-up | 0,87 |

*Note.* RFQY = Reflective Functioning Questionnaire for Youths.

**Table 11**

*SRIS-Y*

| SRIS-Y |  |  |  |  |  |  |  |
| --- | --- | --- | --- | --- | --- | --- | --- |
|  | Score SR |  | RCI SR |  | Score I |  | RCI I |
| Pre | 46 | Pre – post | -3,85* | Pre | 21 | Pre – post | 3,21* |
| Post | 43 | Post – follow-up | -5,13* | Post | 24 | Post – follow-up | 2,14* |
| Follow-up | 39 | Pre – Follow-up | -8,99* | Follow-up | 26 | Pre – Follow-up | 5,35* |

*Note.* SRIS-Y = Self-Reflection and Insight Scale for Youth; * = RCI exceeds (-)1.96.

**Table 12**

*PT-IRI*

| PT-IRI |  |  |  |
| --- | --- | --- | --- |
|  | Score |  | RCI |
| Pre | 23 | Pre – post | -2,53* |
| Post | 15 | Post – follow-up | 1,90 |
| Follow-up | 21 | Pre – Follow-up | -0,63 |

*Note.* PT-IRI = Perspective Taking subscale of the Interpersonal Reactivity Index; * = RCI exceeds (-)1.96.
